# Diversity of coatings on hydrothermal vent gastropods

**DOI:** 10.1038/s41598-025-29638-3

**Published:** 2025-12-02

**Authors:** Agata Bonk, Krzysztof Hryniewicz, Paweł Bącal, Daniel Smrzka, Chong Chen, Crispin T. S. Little

**Affiliations:** 1 Institute of Paleobiology, Polish Academy of Sciences, ul. Twarda 51/55, Warszawa, 00-818 Poland; 2 MARUM, Zentrum für Marine Umweltwissenschaften, 28359 Bremen, Germany; 3 Fachbereich Geowissenschaften, Universität Bremen, 28359 Bremen, Germany; 4 X-STAR, Japan Agency for Marine-Earth Science and Technology (JAMSTEC), 2-15 Natsushima-cho, Yokosuka, 237-0061 Japan; 5 School of Earth and Environment, University of Leeds, Woodhouse Lane, Leeds, LS2 9JT UK

**Keywords:** Deep-sea, Elemental mapping, Gastropod, Hydrothermal vent, Coating, Raman spectra, Environmental sciences, Ocean sciences, Solid Earth sciences

## Abstract

Shells of hydrothermal vent gastropods are often covered by inorganic coatings whose chemical composition, formation mechanisms, and possible function remain poorly understood. We investigated the mineral coatings of six vent gastropod specimens (one each of the species *Lepetodrilus nux*, *Cantrainea jamsteci*, *Desbruyeresia armata*, *Desbruyeresia marisindica*, and two specimens of *Alviniconcha marisindica* from separate vent sites) from four different localities (Iheya North vent field, Okinawa Trough and Myojin-sho Caldera, Izu-Ogasawara Arc in the Pacific Ocean, and Kairei and Edmond vent fields on the Central Indian Ridge) using SEM/EDS and Raman spectroscopy. The resulting data show that the chemical composition of the coatings corresponds to the chemistry of vent fluids at the respective localities and distance from active venting. There was no evidence of mineral replacement of any of the studied shells. In one specimen the coating was partly overgrown by the shell, showing a contemporary growth of the shell and the coating. The texture of the coating on the *Desbruyeresia armata* specimen suggests a possible role of microorganisms in its formation. Gastropod fossils from ancient vent sites are preserved by pyrite, with no remaining carbonate shell. We found pyrite only in coatings of the modern gastropods collected near high-temperature vent sites, indicating that close proximity to active venting may be necessary for successful preservation of vent gastropods in the fossil record.

**Supplementary Information:**

The online version contains supplementary material available at 10.1038/s41598-025-29638-3.

## Introduction

Hydrothermal vents form when seawater percolates through fissures and cracks deep into the ocean crust, where it is heated, reacts with subsurface rocks, and is then convected back to the seafloor and emitted as hydrothermal fluid. These fluids typically have high temperatures (up to 407 °C^1^and contain a mixture of chemically-reduced dissolved minerals and gases. When these fluids mix with the ambient cold seawater, minerals precipitate, producing chimney-like structures and complex mineral mounds (e.g.^2–4^). The majority of hydrothermal vents are found along mid-ocean ridges (65%), followed by back-arc basins (22%), and volcanic arcs (12%)^5^. The geological setting, as well as the type of host rock, and the presence or absence of sediments influence vent fluid composition and the mineralogy of the deposits (Table 1^5^).

**Table 1:** Vent fluid chemistry and deposit mineralogy for different geological settings of hydrothermal systems.

	Geological context	Vent fluid chemistry	Vent deposit mineralogy	References
Structural setting	Mid-ocean ridge	Rich in Fe, Zn, Cu	Pyrite (FeS_2_), chalcopyrite (CuFeS_2_), sphalerite ((Zn, Fe)S), anhydrite (CaSO_4_)	^6^
	Back-arc basin	Rich in Zn, Pb, Ba	Sphalerite dominates; massive sulfides with low Fe content; galena (PbS)	^6^
	Volcanic arc	Rich in Ca, K, Ba	Pb, As, Ba enrichment, native sulfur, pyroclastic rocks	^7^
Host rock	Andesite, rhyolite, dacite	Higher Zn, Pb, As concentrations than in basalt-hosted vents		^8^
	Ultramafic rocks	Higher CH_4_ and H_2_ concentrations than in basalt-hosted vents, presence of Co and Ni		^9^
	Sediment	Metal depletion, enrichment in alakali element, B, NH_4_, organic matter; colder, less acidic, more reduced	Galena, calcite (CaCO_3_), anhydrite, barite (BaSO_4_), silica (SiO_2_)	^10^
Flow regime	Diffuse flow	Fe, Mn, and Si abundance	Fe silicates, Fe and Mn oxides and hydroxides, sulfates, carbonates	^11^

Hydrothermal vents support unique and highly adapted biological communities (e.g.^12–15^). Gastropods are one of the most diverse groups of metazoans in vent environments. They often live on microbial mats and amongst other invertebrates, such as tubeworms and mussels^16^. Some genera, such as *Alviniconcha* in the West Pacific and Indian Ocean, form large colonies on vent chimney walls. Vent gastropods exhibit a range of feeding modes. Most species graze on the surface layer of detritus and bacterial mats^14^. Some, such as *Neomphalus* rely on filter-feeding; certain species of *Lepetodrilus* employ filter-feeding in addition to grazing^17^. Caenogastropod species are largely predators and scavengers^14^. Several gastropod species form symbiotic relations with chemosynthetic bacteria, notable examples being species of two large-bodied genera that can exceed 10 cm in shell height: *Alviniconcha* and *Ifremeria*, which harbor bacteria in their enlarged ctenidia^14^.

Vent fluids are often highly acidic, causing rapid dissolution of calcium carbonate-based molluscan shells^18^. Consequently, modern vent gastropods are commonly found with missing protoconches and with dissolution pits on the adult shell (see Supplementary Figure S1^19–22^). This is especially prevalent in the ontogenetically older areas of the shell where the periostracum has been lost, such as at the apex^23^. Vent gastropods employ various adaptive mechanisms to protect against the corroding environment, such as simplification of shell shape, increasing the shell thickness, and the development of a thick periostracum^14^.

Vent gastropod shells are often found with coatings on their surfaces^21,22,24^. These coatings have the potential to act as protection from shell dissolution, and after the death of the animal also offer a taphonomic pathway for fossilization^18^. Because these coatings obscure shell ornamentation details needed for identification, they are usually physically or chemically removed by taxonomists^25^. Thus, the composition, formation, and possible implications for gastropod biology of shell coatings remain poorly known. In order to provide new knowledge on this topic here we investigate the composition of coatings present on the shell surfaces of a number of vent gastropod species from diverse hydrothermal sites in the Western Pacific and Indian Ocean, and link this to published data on vent fluid and mineral deposit chemistry from the same locations.

## Materials and methods

### Materials

**Fig. 1:**
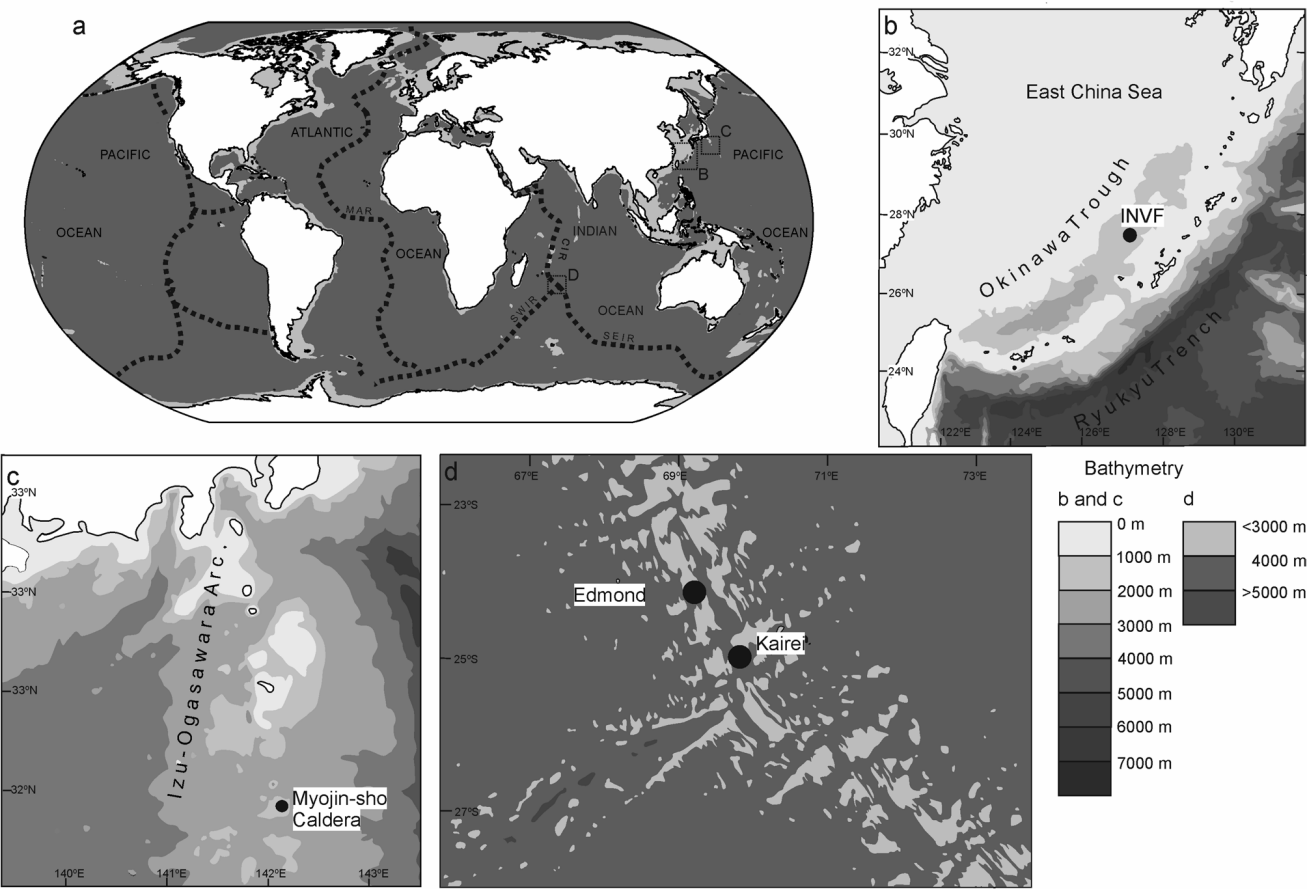
Localization and bathymetry of specimens’ sites of origin; (**a**) localization on the world map; (**b**) bathymetry of the Okinawa Trough; (**c**) bathymetry of the Izu-Ogasawara Arc; (**d**) bathymetry of the Central Indian Ridge.

**Fig. 2:**
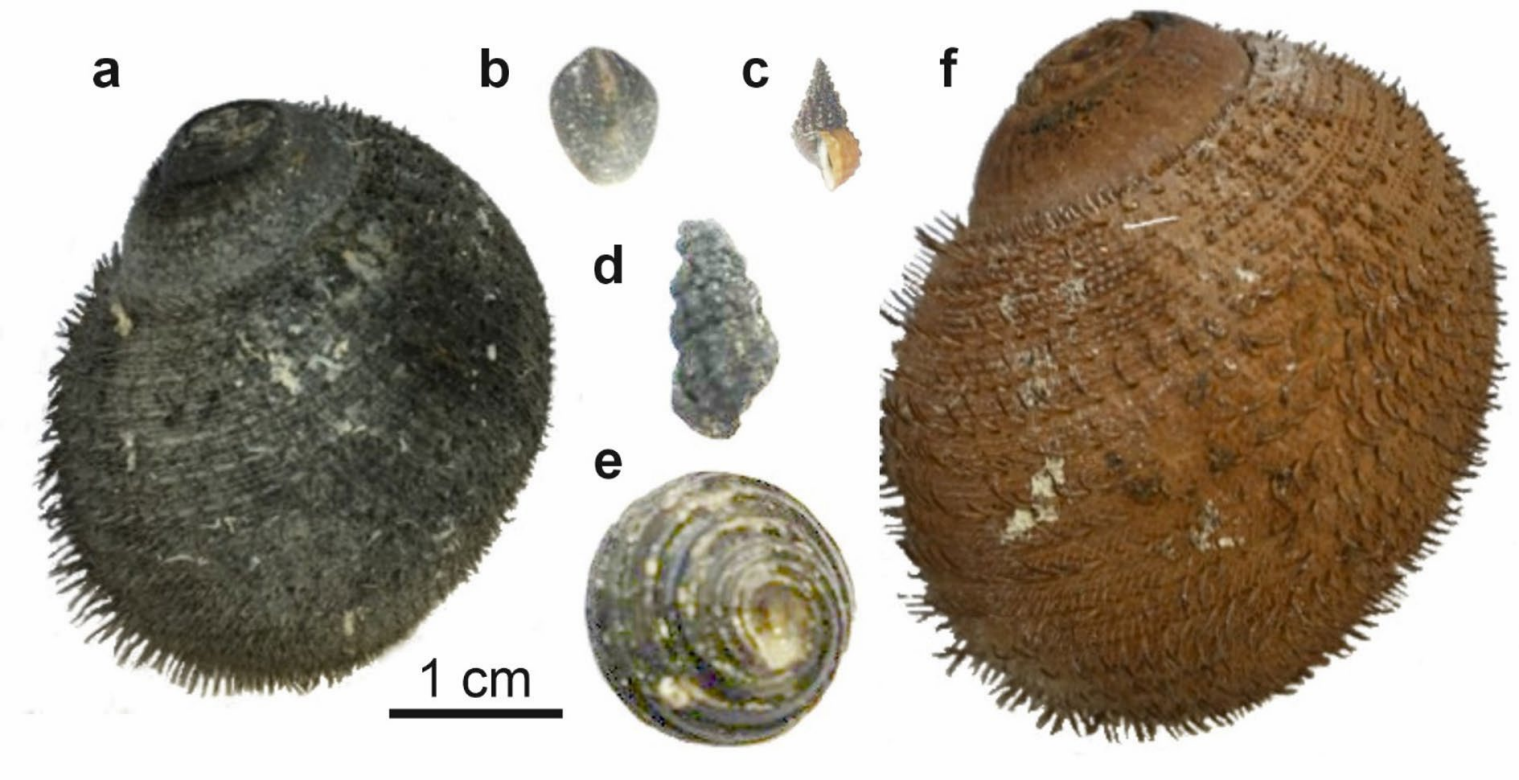
Specimens selected for this study; (**a**) *Alviniconcha marisindica*, ZPAL Ga.22/5, Kairei vent field, Central Indian Ridge, Indian Ocean; (**b**) *Lepetodrilus nux*, ZPAL Ga.22/1, Original Site, Iheya North vent field, Okinawa Trough, Pacific Ocean; (**c**) *Desbruyeresia marisindica*, ZPAL Ga.22/4, Kairei vent field, Central Indian Ridge, Indian Ocean; (**d**) *Desbuyeresia armata*, ZPAL Ga.22/3, Myojin-sho Caldera, Izu-Ogasawara Arc, Pacific Ocean; (**e**) *Cantrainea jamsteci*, ZPAL Ga.22/2, Aki Site, Iheya North vent field, Okinawa Trough, Pacific Ocean; (**f**) *Alviniconcha marisindica*, ZPAL Ga.22/6, Edmond vent field, Central Indian Ridge, Indian Ocean. Scale bar applies to all specimens.

Gastropods were collected alive from four different hydrothermal vent areas (Fig. 1) by suction samplers. The remotely operated vehicle (ROV) *Hyper-Dolphin* was used for sampling from the Original Site (dive #1610) and the Aki Site (dive #1616) of the Iheya North vent field, Okinawa Trough on-board R/V *Kaiyo* cruise KY14-01, as well as the Myojin-sho Caldera (dive #1295) on the Izu-Ogasawara Arc on board R/V *Natsushima* cruise NT11-10 Leg 1; and the human-occupied vehicle (HOV) *Shinkai 6500* was used to sample the Kairei vent field (dives #1450 and #1458) and the Edmond vent field (dive #1457) on the Central Indian Ridge. All submersibles used in this study are owned by Japan Agency for Marine-Earth Science and Technology (JAMSTEC). The gastropod specimens were stored in 99% ethanol upon recovery on-board. Based on initial visual investigation of the species collected, six specimens with the thickest and diversely colored mineral coatings were then selected for detailed study (Table 2; Fig. 2). The six studied specimens are curated at the Institute of Paleobiology, Polish Academy of Sciences, Warsaw, Poland (ZPAL).

**Table 2:** Studied specimens with collection information and geological background data.

Species	Coating	Locality	Ecological setting	Coordinates	Depth [m]	Date collected	Geological and geochemical background
*Lepetodrilus nux* (ZPAL Ga.22/1)	dark grey and thin	Original Site, Iheya North Vent Field, Okinawa Trough (back-arc basin)	hottest part of the North Big Chimney (NBC) mound among *Paralvinella* cf. *hessleri* worm colony	27°47.4556’N 126°53.7987’E	982	12.01.2014	basalt and rhyolite as host rock, terrigenous sediments^26^, fluid temp. ~311 °C, massive sulfides: sphalerite, galena, pyrite; abundant anhydrite after Integrated Ocean Drilling Program (IODP) Expedition 331 at the NBC mound in 2011^27^
*Cantrainea jamsteci* (ZPAL Ga.22/2)	black and thin	Aki Site, Iheya North Vent Field, Okinawa Trough (back-arc basin)	low-temperature diffuse flow among bathymodiolin mussel colony	27°46.1298’N 126°54.1588’E	1087	25.01.2014	
*Desbruyeresia armata* (ZPAL Ga.22/3)	black and thick, obscuring ornamentation details	Myojinsho Caldera, Izu-Ogasawara Arc (volcanic arc)	diffuse flow among mussel colony	31°53.012’N 139°58.203’	855	30.06.2011	caldera floor: pumice fragments, volcanic glass, Mn- and Fe-consolidated sediments; mineral deposits: barite, sphalerite, galena; deposits covered by Fe and Mn oxyhydroxide films^28^
*Desbruyeresia marisindica* (ZPAL Ga.22/4)	black, medium thickness	Kairei Vent Field, Central Indian Ridge (mid-ocean ridge)	wall of an active chimney, among *Archinome jasoni* worm colony	25°19.2249’S 226°2.4123’E	2434	14.02.2016	ultramafic host rock, vent fluids: temp. 315–365 °C, high Fe/Mn ratio^29^, mineral deposits: chalcopyrite, other Cu-rich sulfides, sphalerite, pyrite, Co and Ni enrichment^30^
*Alviniconcha marisindica* (ZPAL Ga.22/5)	dark grey and thin	Kairei Vent Field, Central Indian Ridge (mid-ocean ridge)	hottest part of the Monju Chimney, colony living alongside *Rimicaris kairei* shrimp	25°19,2298’S 226°24143’E	2423	27.02.2016	
*Alviniconcha marisindica* (ZPAL Ga.22/6)	reddish and thin	Edmond Vent Field, Central Indian Ridge (mid-ocean ridge)	diffuse flow at the active ‘Shrimp Castle’ chimney, colony living amongside *Bathymodiolus marisindicus* mussels	23°52,6621’S 226°35,7959’E	3279	26.02.2016	vent fluids: temp. 381 °C, Fe concentration two times higher than at Kairei^31^ SiO_2_ and Fe enrichment linked to microbial activity^32^; widespread reddish Fe oxyhydroxide sediments hosting microbial mats and coating mineral deposits^29^; mineral deposits: sphalerite, pyrite, marcasite, chalcopyrite, anhydrite, barite^33^.

## Methods

For the four smaller specimens (one each of the lepetodrilid limpet *Lepetodrilus nux*, the abyssochrysoidean snails *Desbruyeresia marisindica* and *Desbruyeresia armata*, and the colloniid snail *Cantrainea jamsteci*) all remaining soft tissues were removed prior to further preparation. The *C. jamsteci* specimen was then longitudinally sectioned using a Dremel rotary cutter. This tool was also used to remove roughly 10 mm pieces of shell from the apertural margins of each of the two large *Alviniconcha marisindica* specimens from two different Indian Ocean vent fields (Supplementary Fig. S2). One piece was removed from the specimen from Kairei vent field and two pieces from the specimen from Edmond vent field. All six shell samples were then critically point dried (CPD) using ethanol at the University of Leeds, UK, in order to minimize the detachment of the coatings from the shells during subsequent preparation. However, the CPD process caused the *A. marisindica* samples to curl in an external shell direction (see Results), most likely due to greater contraction of the thick periostracum compared to the underlying thin shell. It was therefore not possible to obtain external shell surface views of either of the *A*. *marisindica* specimens using scanning electron microscopy (SEM).

For the other post-CPD specimens the shell surfaces were analyzed by SEM with energy dispersive X-ray spectrometry (EDS). This was done using a Thermo Fisher Quattro S SEM in the Institute of Paleobiology, Polish Academy of Sciences, Warsaw, Poland. This environmental SEM was equipped with an Octane Elect (EDAX) EDS detector. Elemental composition measurements were performed at the accelerating voltage of 15 kV. Whilst quantitative analysis using SEM/EDS is possible, it can prove problematic when working with topographically complex surfaces, such as biological samples^34^. Therefore, absolute quantitative values were not used here for describing the abundance of each element in the sample. Each element emits X-rays with characteristic energies, unique to that element. However, due to similar values of those energies some elements, e.g. Pb and S < Kα peak (2.3 keV) and the Pb Lα peak (2.34 keV) or Bi Lα peak (2.42keV) >, may be problematic to quantify or even to distinguish, which is partially the case encountered in this study. Deconvolutions of such signals (and following elemental mapping) are especially difficult when the signal is broad and has a low signal to background ratio which can occur i.e. with low content in the sample, low conductivity of the sample, or with a rough and non-compact sample^35,36^.

In order to determine the interface between the mineral coatings and the gastropod shells, each specimen was set into epoxy resin (Araldite 2020) blocks, which were then ground down or cut to produce cross-sections of the shells. The resin blocks were then polished, coated with a 10 nm thick layer of carbon using CCU-10 sputter. The samples were imaged using SEM/EDS. The SEM was equipped with a Backscattered Electrons (BSE) detector and operated at accelerating voltage of 10 kV. To keep high resolution of samples a large area imaging was performed using MAPS3 software (Thermo Fisher Scientific; https://www.thermofisher.com/pl/en/home/electron-microscopy/products/software-em-3d-vis/maps-software.html#features), which allows for automated images collection and stitching. The identification of certain minerals in the coatings was based on the co-occurrence of elements (Table 3), supported by geochemical data from the respective collection site, and Raman spectroscopy.

**Table 3:** Combinations of elements from elemental maps and minerals they likely form based on their respective geochemical composition.

Combination of elements	Putative minerals	Minerals confirmed by Raman spectroscopy
Ca, O, S	Anhydrite	Inconclusive
Fe, S	Pyrite	Pyrite
Fe, S, Cu	Chalcopyrite	Inconclusive
Fe, S, Zn	Sphalerite	Inconclusive
Pb, S	Galena	Inconclusive
Ba, O, S	Barite	Inconclusive
Mn, O	Manganese oxides	Likely todorokite, romanechite, hollandite
Fe, O	Iron oxides	Inconclusive
Si, O	Silica	Inconclusive
Fe, Si, O	Iron silicates	Inconclusive

The Raman spectroscopy was performed on the polished samples embedded in resin blocks using a Renishaw InVIa Raman spectroscope at the Institute for Mineralogy, University of Bremen. A Renishaw RL532C class 3B continuous wave laser (532 nm wavelength) operating in high confocality mode was used. Exposure time was set to 10 s using 10% laser power (24 mW), with a grating set at 1800 Lmm^− 1^. The spectroscope was coupled to a WiRE© 5.5 (Renishaw’s Windows(r)-based Raman Environment (WiRE) software, version 5.5.; https://www.renishaw.com/en/raman-software-−9450?srsltid=AfmBOoqFPrxywO8jvC7k79ufBSXCqC8TLnuGNKC7U_aoZS3Y2in_1g-q) software for laser and camera control, and for raw data processing.

The samples were not analyzed by X-ray diffraction (XRD) due to the small amount of the material available for study.

## Results

### *Lepetodrilus nux;* Iheya North vent field, Okinawa Trough

**Fig. 3:**
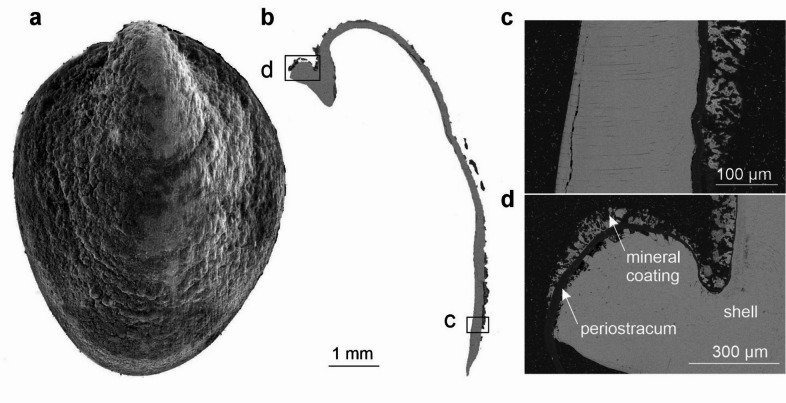
*Lepetodrilus nux*, ZPAL Ga.22/1, Original Site, Iheya North vent field, Okinawa Trough, Pacific Ocean; (**a**) SEM surface view (**b**) SEM BSE view of the cross-section through the shell. Lettered insets in (**b**) correspond to images (**c**) and (**d**).

EDS elemental maps have been generated for five areas of the shell surface of *L. nux* and for four areas of the cross-section through the shell (Fig. 3; Supplementary Fig. S3). The coating is visible as a porous structure on SEM BSE images of the cross-section through the shell. It is present on the surface of the periostracum, which appears as a grey tissue between the brighter coating and shell (Fig. 3). The coatings appear as frondose, diffuse textures attached to the periostracum (Fig. 3b–d). EDS elemental maps of the shell surface of *L. nux* show a rather even distribution of C, N, and O, as well as Ba and Fe, which are less abundant. Mn, P, S, and Ca constitute the most abundant elements in the coating. While the first two are distributed rather evenly on the surface of the shell, S and Ca co-occur in patches of high intensities. Pb or Bi, if present, frequently follows this pattern, although it exhibits less intensity (Supplementary Fig. S4). In the cross-section of the shell, N and S are most abundant in the periostracum. S can also be observed in the coating, along with abundant P and O. Ca and O are present inside the shell (Supplementary Fig. S5).

Raman spectra of coatings collected from the *L. nux* specimen are too ambiguous to extract reliable mineralogical information, showing diffuse peaks between 630 and 640 cm^− 1^ (Supplementary Fig. S6a). Most of the obtained spectra from all of the studied species also show similar, broad “humps” between 1200 and 1600 cm^− 1^e.g. Figs. S6b-c, S12, S21, S26. A test measurement was conducted on the resin in which *D. marisindica* is embedded in order to detect a possible influence of artificially-derived resin material, which has characteristic and distinguishable Raman spectrum (Supplementary Fig. S7).

### *Cantrainea jamsteci;* Iheya North vent field, Okinawa Trough

**Fig. 4:**
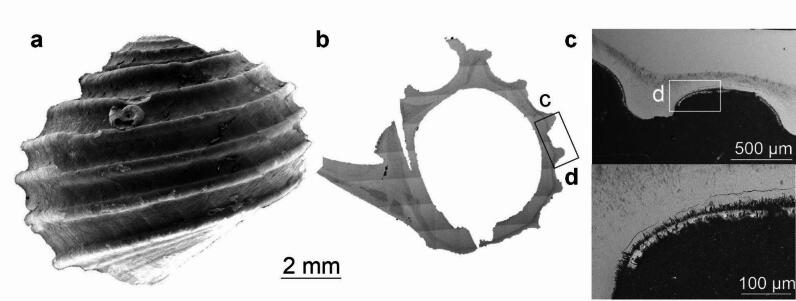
*Cantrainea jamsteci*, ZPAL Ga.22/2, Aki Site, Iheya North vent field, Okinawa Trough, Pacific Ocean; (**a**) SEM surface view (**b**) SEM BSE view of the cross-section. Lettered inset in (**b**) corresponds to image (**c**) and lettered inset in (**c**) corresponds to image (**d**).

Four areas of the shell surface of *C. jamsteci* and four areas of the cross-section (Fig. 4; Supplementary Fig. S8) have been mapped by EDS. The coating is visible on SEM BSE images of the cross-section as a thin µm-thick veneer (Fig. 4b), that does not cover the entire surface of the shell (Fig. 4b–d). C, N, and O are evenly distributed over the shell surface. Ba is present in one out of four areas studied, showing even distribution and moderate abundance. S and Ca are abundant (Supplementary Fig. S9); however, their patterns of distribution are slightly similar only in Area 2 of the shell. Bi or Pb, if present are rather evenly distributed but its abundance is slightly higher in areas of higher S abundance (Supplementary Fig. S10). Mn and Fe are moderately abundant and rather evenly distributed. In the Area 2 of the shell Mn seems to be present, where S is absent (Supplementary Fig. S10), however, this pattern is not observed for other areas. Even though the distribution of Fe does not follow the S distribution pattern, one spot of high Fe, S, and Bi or possibly Pb abundance can be observed (Supplementary Fig. S10). On EDS elemental maps of the cross-section the shell is composed mainly of Ca and O. The latter is slightly more abundant in the coating. N is present only in trace amounts, but more abundant in the periostracum (Supplementary Fig. S11). S and P also exhibit higher abundance in the coating (Supplementary Fig. S11), but their abundance is low in general (S is present only in one area of the shell). Mn is the most abundant element present in the coating (Supplementary Fig. S11). Raman spectrum of coatings obtained from *C. jamsteci* features an unclear, broad peak at ~ 1550 cm^− 1^ (Supplementary Fig. S12), from which we cannot determine reliable mineralogical information.

### *Desbruyeresia armata*; Myojin-sho Caldera, Izu-Ogasawara Arc

**Fig. 5:**
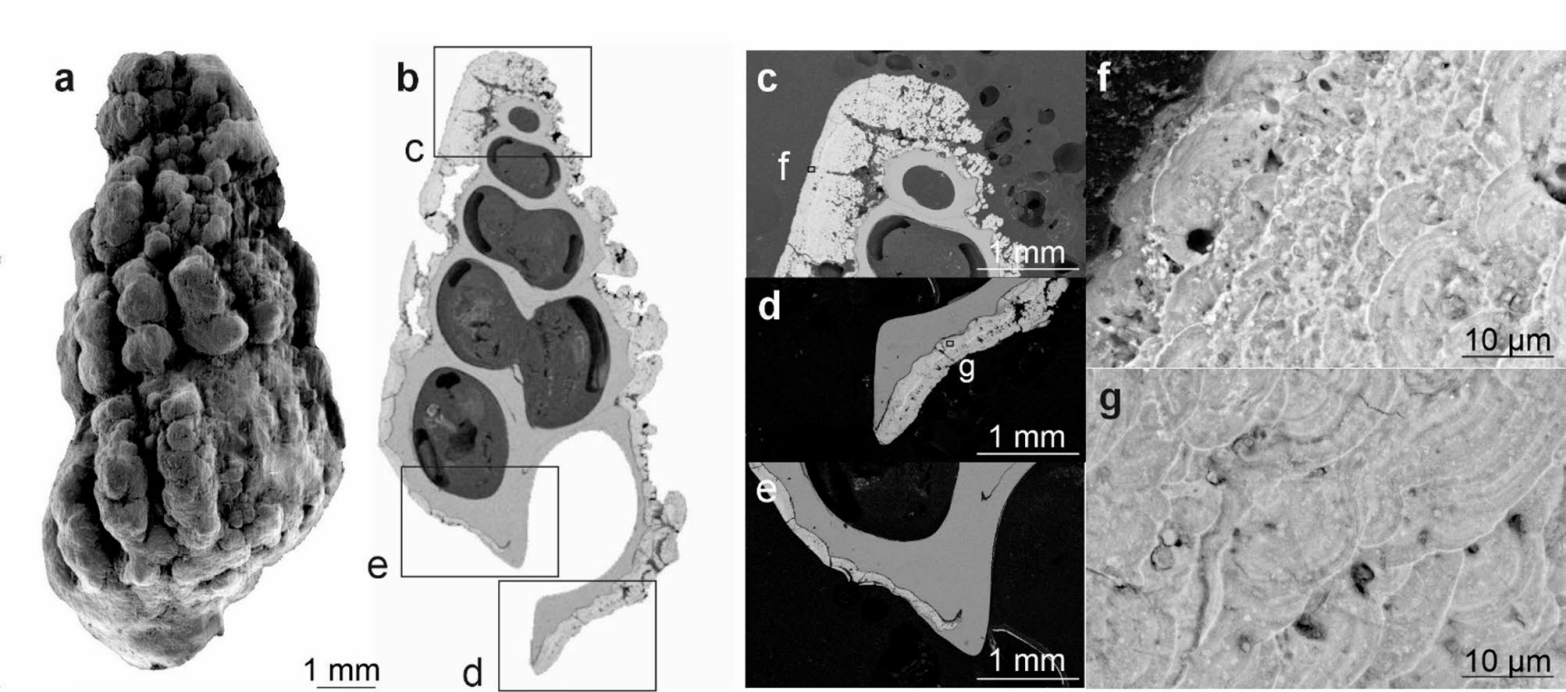
*Desbruyeresia armata*, ZPAL Ga.22/3, Myojin-sho Caldera, Izu-Ogasawara Arc, Pacific Ocean; (**a**) SEM surface view (**b**) SEM BSE view of the cross-section. Lettered insets in (**b**) correspond to images (**c**), (**e**), and (**d**). Lettered inset in (**c**) corresponds to image (**f**) and lettered inset in (**d**) corresponds to image (**g**).

**Fig. 6:**
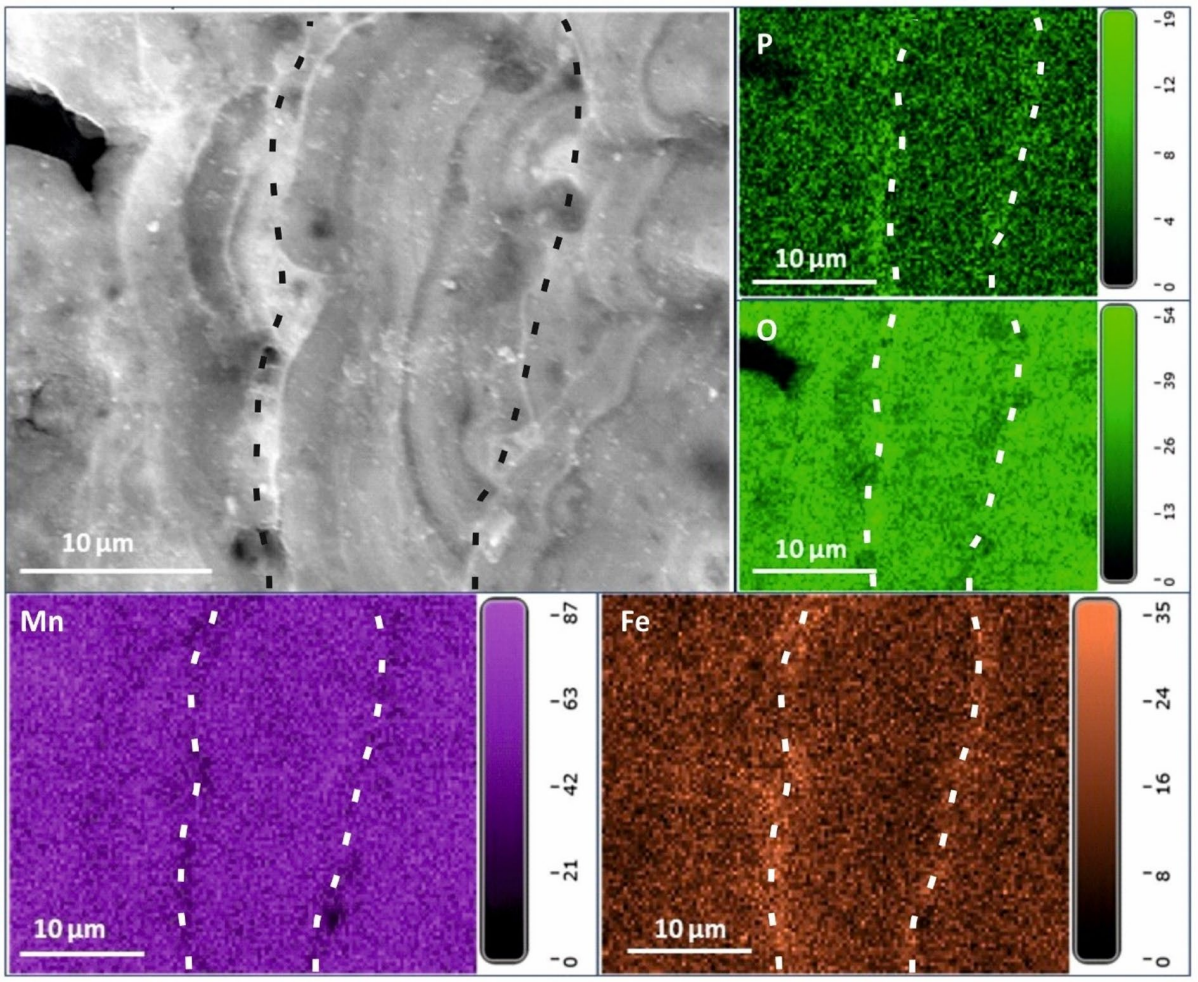
EDS elemental maps of Area 13 of the cross-section through the shell of *Desbruyeresia armata*, ZPAL Ga.22/3, Myojin-sho Caldera, Izu-Ogasawara Arc, Pacific Ocean. Top left shows the field of view; with the remaining showing the abundance of P, O, Fe and Mn as indicated. Dashed lines indicate bands of higher or lower abundance.

EDS elemental maps have been generated for six areas of the shell surface of *Desbruyeresia armata* and for seven areas of the cross-section (Fig. 5; Supplementary Fig. S13). The cross-section through *D. armata* shows the presence of a thick coating on the surface of the shell, which largely obscures the ornamentation details (Fig. 5b). This coating is considerably thicker than in *C. jamsteci* and *L. nux*, reaching almost 1 mm in thickness (Fig. 5b–d). At the tip of the apertural opening the mineral coating is partly overgrown by the calcareous shell (Fig. 5e). At higher magnification the coating has a distinct colloform texture with distinct, µm-thick laminations (Fig. 5f-g). Both SEM images and EDS elemental maps generated under higher magnification show alternating bands of higher Fe abundance that correspond to bands with higher P and lower Mn and O abundances (Fig. 6).

EDS elemental maps of the shell surface of *Desbruyeresia armata* show even distribution of moderately abundant C and N. O is highly abundant and also evenly distributed. Cu is evenly distributed over the shell surface and moderately abundant. Co appears in trace to moderate amounts and exhibits even distribution. S and Pb (or Bi) are abundant and rather evenly distributed, however, they also appear in spots of higher abundance that follow a similar pattern. Less abundant Zn frequently co-occurs with S and Pb (Supplementary Fig. S14). Mn and Fe form a dense and rather homogenous cover (Supplementary Fig. S15) with Fe occasionally forming spots of higher abundance together with S, Pb and Cu (see Supplementary Figure S16). Mn seems to be less abundant or absent in areas of higher C abundance (Supplementary Fig. S15). EDS elemental maps of the cross-section of the shell show low abundance of C and N. O is more abundant in the coating than in the shell. Similar pattern can be observed for Zn and S. Cu, and Pb are also slightly more abundant in the coating, however, their abundance is low in general. The distribution of Si is spotty and its abundance is very low. Ca is present in high abundance in the shell. The abundance of Fe is rather moderate and higher in the coating than in the shell. Mn is the most abundant metal in the coating and is almost completely absent from the shell.

**Fig. 7:**
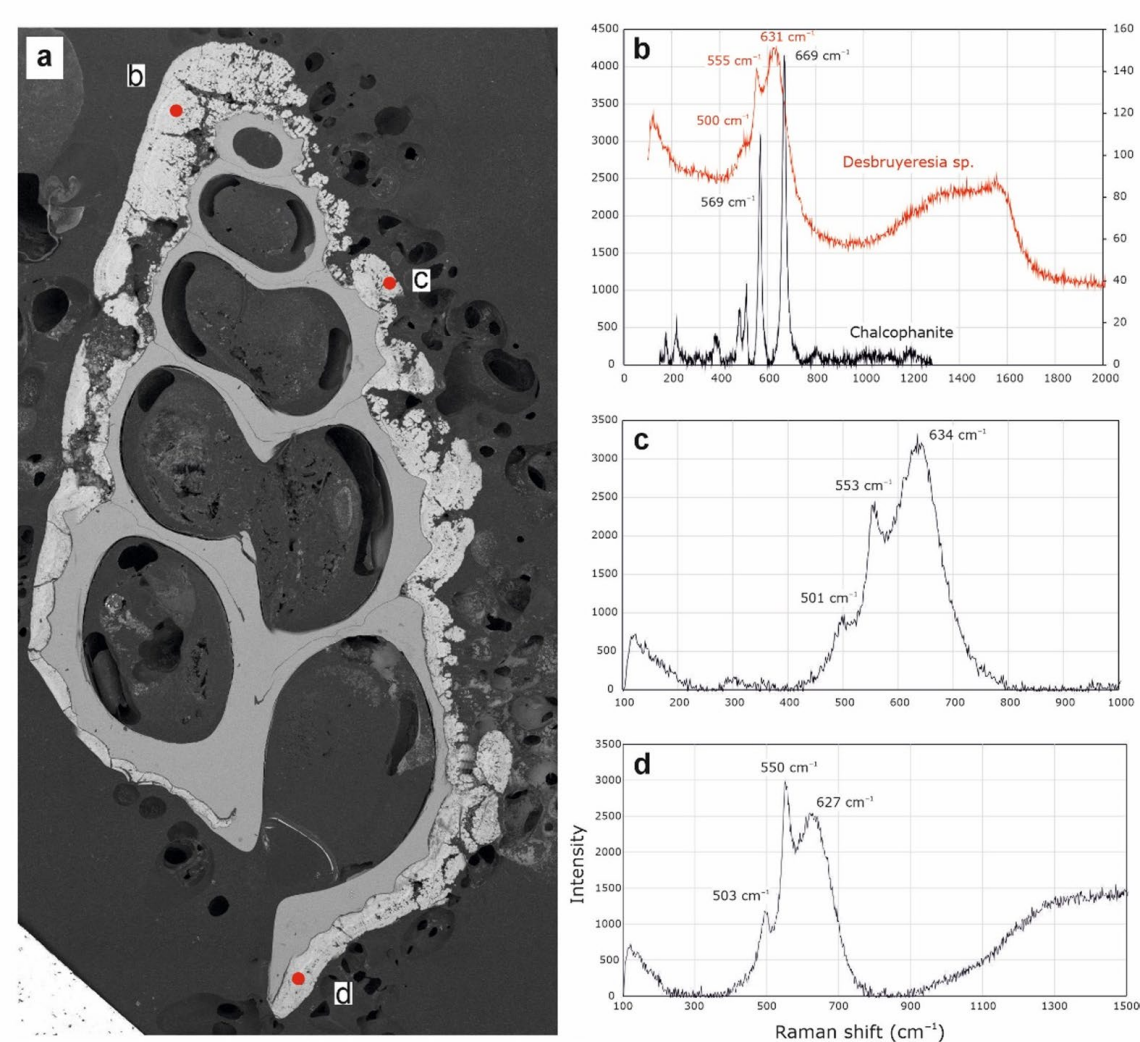
Raman spectra of *Desbruyeresia armata*, ZPAL Ga.22/3, from three locations of the specimen highlighted in (**a**) corresponding to (**b**), (**c**), and (**d**).

Raman spectra from *Desbryueresia armata* show spectra with peaks between 500 and 630 cm^− 1^, and diffuse peaks of varying width between 630 and 640 cm^− 1^, as well as between 690 and 720 cm^− 1^ (Fig. 7).

### *Desbruyeresia marisindica;* Kairei vent field, Central Indian Ridge

**Fig. 8:**
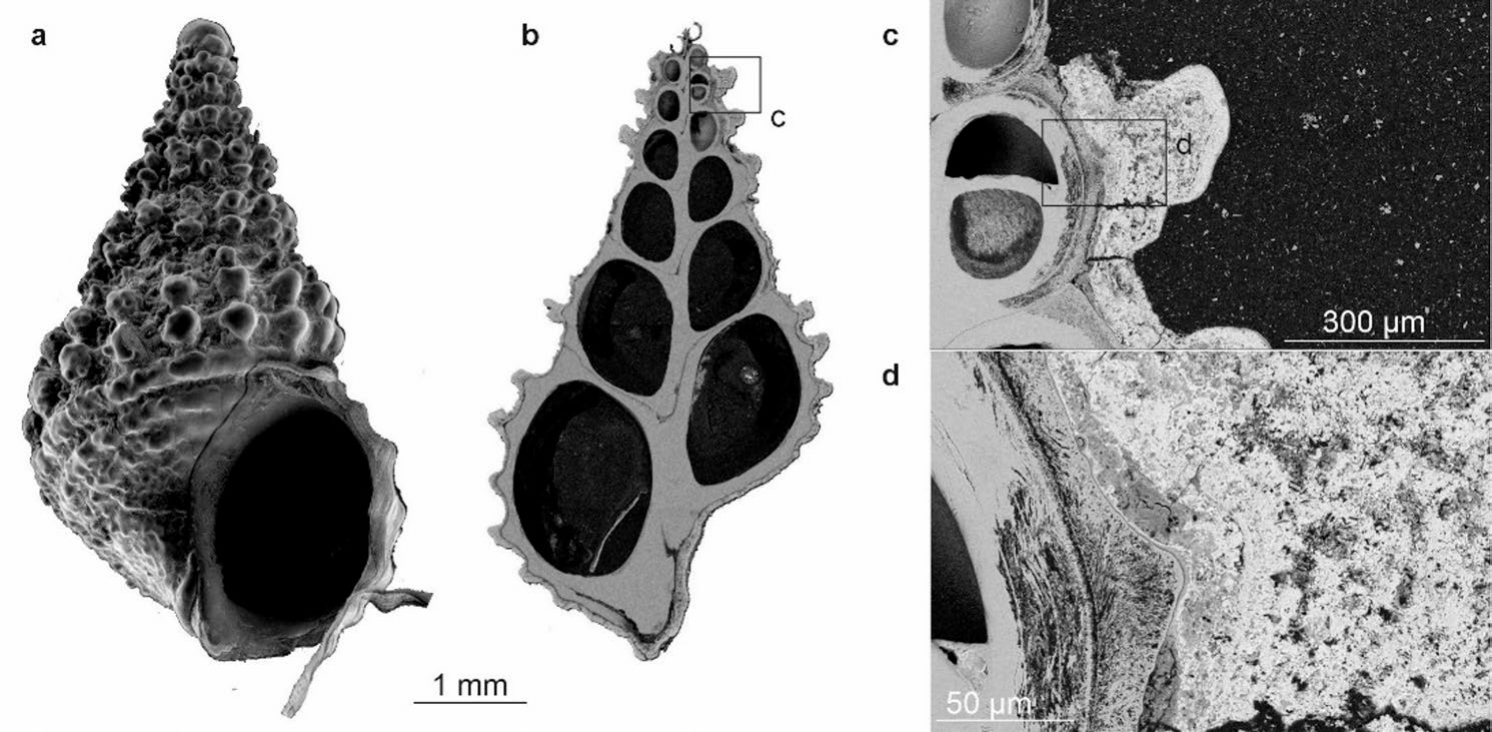
*Desbruyeresua marisindica*, ZPAL Ga.22/4, Kairei vent field, Central Indian Ridge, Indian Ocean; (**a**) SEM surface view (**b**) SEM BSE view of the cross-section through the shell. Lettered inset in (**b**) corresponds to image (**c**) and lettered inset in (**c**) corresponds to image (**d**).

**Fig. 9:**
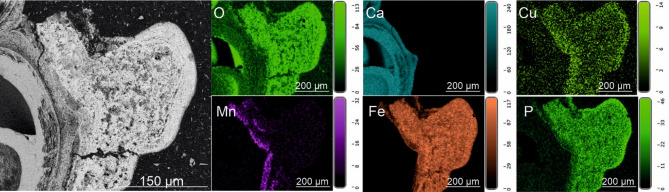
EDS elemental maps of Area 11 of the cross-section through the shell of *Desbruyeresia marisindica*, ZPAL Ga.22/4, Kairei vent field, Central Indian Ridge, Indian Ocean. Left shows the field of view. The distribution and abundance of O, Ca, Cu, Mn, Fe and P shown on the right.

EDS elemental maps have been generated for nine areas of the shell surface of *D. marisindica* and for seven areas in total of the shell cross-section (Fig. 8; Supplementary Fig. S17). SEM BSE images of the cross section show that the morphology of the coating corresponds largely to the ornamentation of spiral ribs and axial nodes, and is thickest on the nodes (Fig. 8c, d). When viewed under higher magnification, the coating is composed of two parts: a thin, more homogenous outer layer and a thicker, more porous layer directly in contact with the shell (Fig. 8d). EDS elemental maps of the shell surface of *D. marisindica* show a moderate and even distribution of C. P and Si are also evenly distributed but slightly more abundant, whilst O is the most abundant element. Even distribution and relatively low abundance can be observed for Mn, and possible traces of Co, which are not present in all nine areas of the shell studied. Trace amounts of Zn can be observed in one area of the shell. S and Ca are relatively abundant, compared to other elements, and exhibit rather even distribution, with spots of higher abundance, which sometimes co-occur. In one place, S forms a spot of higher abundance with Cu (Supplementary Fig. S18). Fe forms a rather dense homogenous cover and is the most abundant metal present in the coating. However, spots where Fe is less abundant or almost completely absent, are present. These spots seem to co-occur with spots of higher S and Ca abundance (Supplementary Fig. S19). EDS elemental maps of the cross-section show an abundance of Fe, O, Si, and P in the mineral coating (Supplementary Fig. S20; Fig. 9). Cu and S are slightly more abundant in the coating than in the shell, while Ca occurs exclusively in the shell (Fig. 9). Mn is also present in the coating; however, a greater abundance of this element is present at the shell-coating boundary (Fig. 9). Cu, although low in abundance, seems to be slightly more abundant in the outer coating layer (Fig. 9). Raman spectra from *D. marisindica* show several diffuse peaks around 700 cm^− 1^, as well as two peaks at approximately 1350 and 1566 cm^− 1^ (Supplementary Fig. S21). The test spectrum for resin obtained from this sample shows a very distinct pattern different from any other obtained from the coatings, with sharp peaks at 1465, 1603, 2870, 2920, and 3070 cm^− 1^ (Supplementary Fig. S7).

### *Alviniconcha marisindica*, Kairei vent field, Central Indian Ridge

**Fig. 10:**
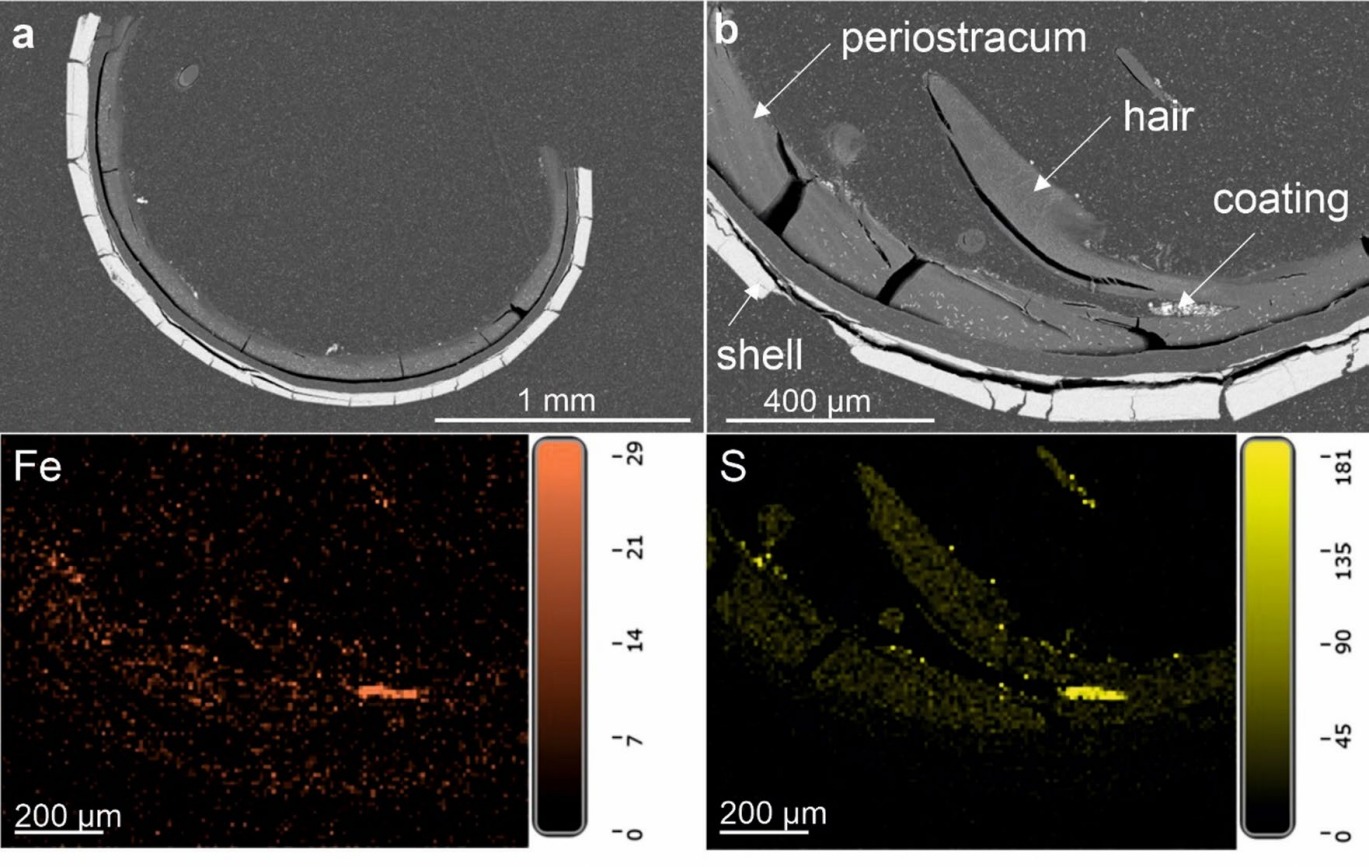
SEM BSE images of the two cross-sections through the shell of *Alviniconcha marisindica*, ZPAL Ga.22/5, from the Kairei vent field, Indian Ocean. Bottom EDS elemental maps of the cross-section (**b**). Distribution and abundance of Fe and S in (**b**) shown below.

EDS elemental maps have been created for five areas of the shell surface of *A. marisindica* from the Kairei vent field and for four areas in total of the two cross-sections through the shell (Fig. 10; Supplementary Fig. S22). The thick and hairy periostracum is visible on SEM BSE images of the cross-section. The coating is barely visible but more concentrated in certain areas (Fig. 10; Supplementary Fig. S22). EDS elemental maps of the shell surface of *A. marisindica* show abundant and evenly distributed cover of C, N, and O. Ca is abundant but appears as numerous spots of higher concentration. S and Fe occur in high abundance; their distribution is rather uneven and frequently follows the same pattern. Cu is less abundant than S and Fe but often appears in the same areas (Supplementary Fig. S23). Traces of Co, if present, follow the distribution of Fe (Supplementary Fig. S23, S24). Zn appears in higher than trace amounts only in one area of the shell, where it co-occurs with S. Interestingly, Fe and Cu appear to be less abundant at this spot (Supplementary Fig. S24). EDS elemental maps of the cross-sections through the shell show an abundance of C and N in all the studied areas of the periostracum. O is more abundant in the shell and the coating than in the periostracum. Ca is present almost exclusively in the shell. S is highly abundant in the coating, where it forms areas of higher concentration, which usually co-occur with less abundant Fe (Fig. 10). The other cross-section (Fig. 10a) differs in apparent absence of Fe on EDS maps. The area where Fe and S coincide show Raman spectra with two small but sharp peaks at 351 and 367 cm^− 1^, and one larger peak at 1535 cm^− 1^, respectively (Supplementary Fig. S25).

### *Alviniconcha marisindica*, Edmond vent field, Central Indian Ridge

**Fig. 11:**
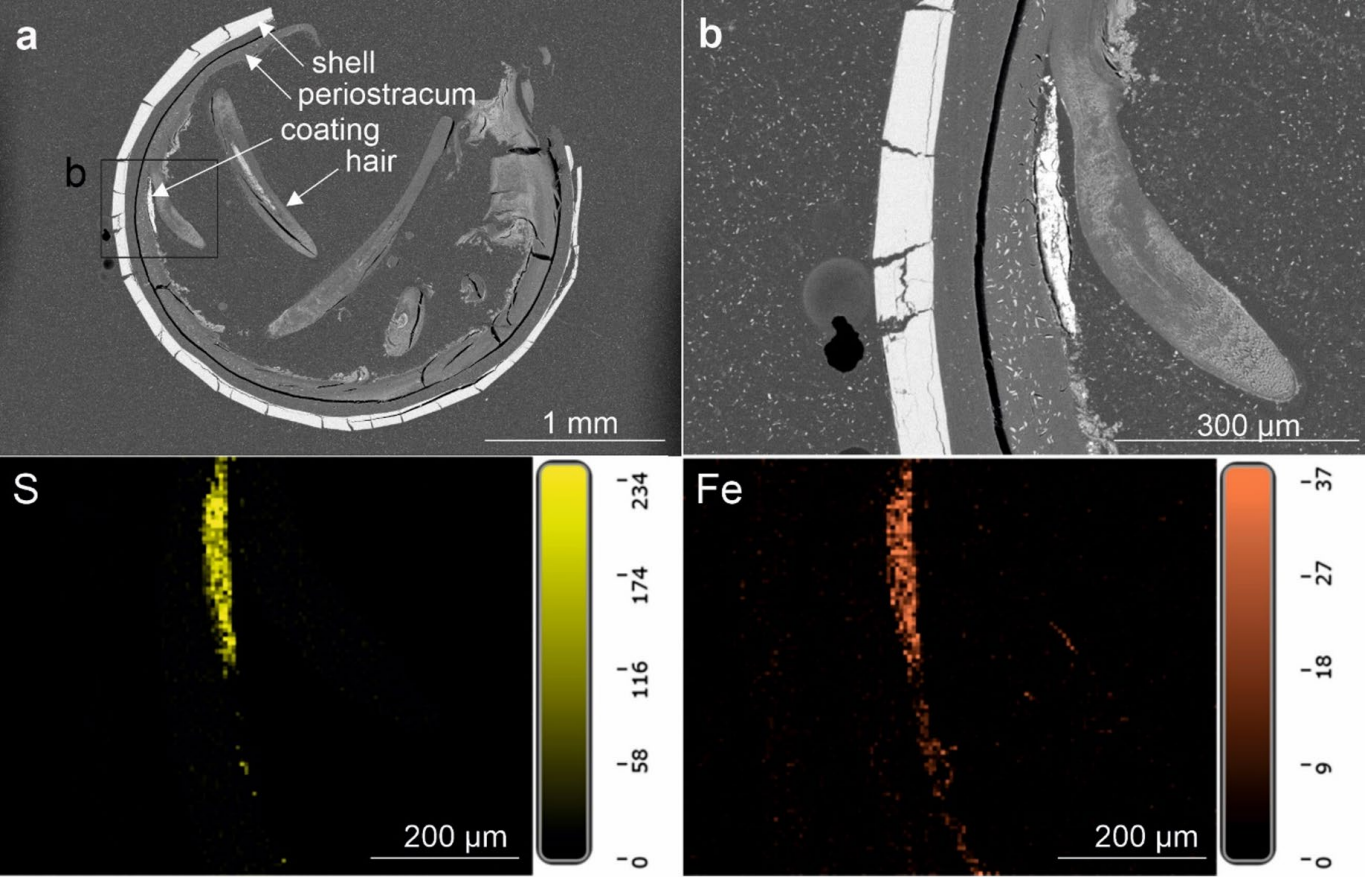
SEM BSE images of the cross-section through the shell of *Alviniconcha marisindica*, ZPAL Ga.22/6, from the Edmond vent field, Indian Ocean. Bottom EDS elemental maps of the cross-section (**b**). Distribution and abundance of Fe and S in (**b**) shown below.

**Fig. 12:**
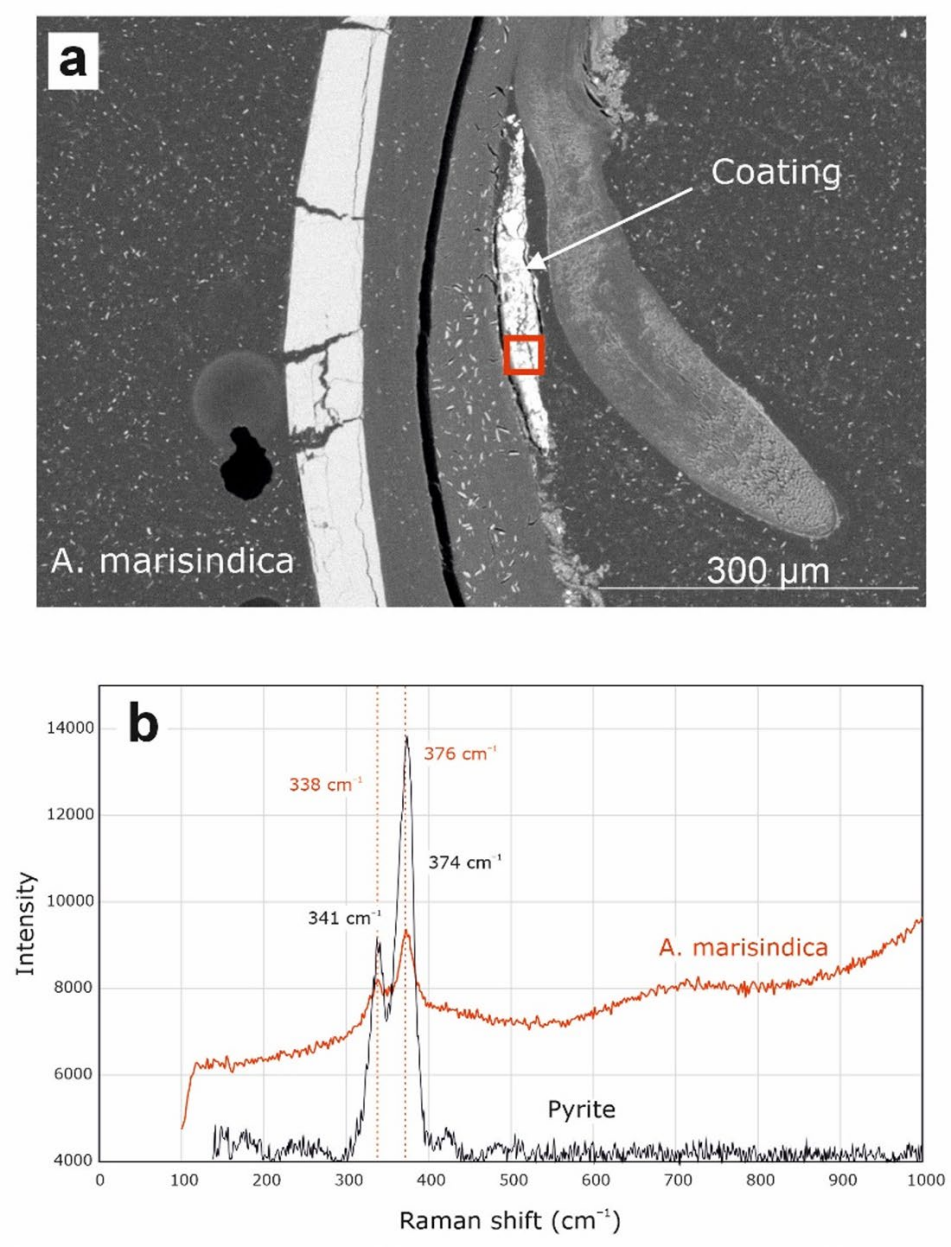
Raman spectrum from *Alviniconcha marisindica*, ZPAL Ga.22/6, Edmond vent field, Indian Ocean (**a**) suggesting the presence of pyrite (**b**).

EDS elemental maps have been created for five areas of the shell surface of *A. marisindica* from the Edmond vent field and for three areas of the cross-section through the shell (Fig. 11; Supplementary Fig. S26). SEM BSE images of the cross-section through the shell show the presence of a thick and hairy periostracum, as well as a thin mineral coating (Fig. 11; Supplementary Fig. S26). EDS elemental maps of the shell surface of *A. marisindica* from the Edmond vent field show abundant and evenly distributed C, N, O, Ca and Si. The latter two elements occasionally form spots of higher abundance. Zn is present in low abundance and, depending on the area of the shell, exhibits even or spotty distribution. Ba appears in two areas of the shell out of five studied. Fe is abundant and forms a homogenous cover on the surface of the shell (Supplementary Fig. S27; S28; S29), which seems to occasionally co-occur with very abundant Si (Supplementary Fig. S28). S is rather abundant but its distribution is uneven. Although less abundant, Bi often exhibits similar pattern of distribution (Supplementary Fig. S27, S29). In one example S and Pb or Bi occur together with Cu (Supplementary Fig. S29). EDS elemental maps of the cross-section show even distribution and relatively high abundance of C, N, and O. Ca occurs in the shell. S exhibits high abundance and rather even distribution, while forming areas of higher concentration with less abundant Fe (Fig. 11). One Raman spectrum from *A. marisindica* reveals two distinct peaks at roughly 340 and 376 cm^− 1^ (Fig. 12). A further Raman spectrum obtained slightly offset from the first spectrum shows two sets of distinct peaks between 338 and 377 cm^− 1^, and between 1400 and 1566 cm^− 1^ (Supplementary Fig. S30).

## Interpretation and discussion

### *Lepetodrilus nux* and *Cantrainea jamsteci*, Iheya North vent field, Okinawa Trough

The occurrence pattern of the most abundant elements in the coating of *Lepetodrilus nux* suggests that it may consist primarily of Ca sulfate and Mn oxides, as well as slightly less abundant Pb sulfides, Ba sulfates, and Fe oxides (Fig. 3; Supplementary Fig. S4). Since the studied specimen of *L. nux* comes from the hottest part of the NBC mound in 2014, the presence of Ca sulfate (provisionally interpreted here as anhydrite; Table 3) is not surprising. The drilling expedition of 2011^27^ resulted in the rapid formation of small chimney structures formed of Ca sulfates, galena, chalcopyrite, and sphalerite^37^. We do not observe either of the latter two in the coating of *L. nux*, as only trace amounts of Cu and Zn were present. The occurrence of abundant Pb is characteristic for back-arc hydrothermal systems^6^, as well as for vent fields covered with terrigenous sediments^9^. Barite is also frequently found at back-arc settings^6^, which is in agreement with detection of Ca sulfate in coatings of *L*. *nux* from Iheya North vent field. Abundant Mn oxides are associated with P, which is likely scavenged as PO_4_^3−^ from the surrounding seawater. Less abundant Fe has a slightly similar pattern of distribution as putative Mn oxides, which suggests it likely occurs in the form of Fe oxides.

In contrast to *L. nux*, the EDS elemental maps of the coating of *Cantrainea jamsteci* point to absence of Ca sulfates (Fig. 4, Supplementary Fig. S9). Ca has a different pattern of distribution than S and often occurs in areas where S is absent (Supplementary Fig. S9). These sites of high Ca abundance may represent places where periostracum has been lost, especially since other elements are also absent or significantly less abundant in these areas. The apparent lack of Ca sulfates in the coating of *C. jamsteci* may be explained by the fact that the specimen was collected from an area of low-temperature diffuse flow. It is also consistent with abundant possible Mn oxides, present on the shell surface, which often precipitate at diffuse flows, further away from hot and reducing conditions^11^, or form as a result of hydrothermal plume fall-out^38^. As in the case of *L. nux*, the coating of *C. jamsteci* is likely enriched in Ba, K, and sulfate. However, Bi or Pb is moderately abundant in the *C. jamsteci* coating and exhibits a distribution pattern similar to S and Fe (Supplementary Fig. S10). Bi enrichment is common for pyrites from hydrothermal systems covered with sediments^39^, as well as for ones hosted by felsic basement rocks^40^.

Zn is often enriched in vent fluids from hydrothermal systems at back-arc basins or with sedimentary component^40^. Massive sulfides from the Iheya North vent field are also significantly enriched in Zn^41^. However, Zn is present in the coating of *L. nux* only in trace amounts and is completely absent from the shell surface of *C. jamsteci*, which might be due to the relatively low temperature in their environment.

### *Desbruyeresia armata*, Myojin-sho Caldera, Izu-Ogasawara Arc

The coating of *Desbruyeresia armata* consists mainly of homogenous Mn and Fe (Fig. 5; Supplementary Fig. S15). Mn and Fe commonly occur in the Myojin-sho Caldera in the form of consolidated sediments and oxyhydroxide films, covering massive sulfides^28^. They are interpreted as a late-stage hydrothermal product^28,42^ and can originate both from the plume fall-out, as well as from precipitation near the low-temperature diffuse flows, where our specimen was collected. Mn and Fe deposits from the area of the Izu-Ogasawara Arc often have both hydrothermal and hydrogenetic origin, however, in proximity to active volcanoes hydrothermally derived Mn and Fe deposits are dominant^42^.

Raman spectra obtained from *Desbruyeresia armata* all point towards the presence of varying forms of Mn oxide minerals. The three spectra are characterized by two large and one small peak at slightly different positions within the spectrum (Fig. 7b–d), as well as one large peak at ~ 630 cm^− 1^. The prominent, yet often diffuse peaks observable between 600 and 700 cm^− 1^ are present in other specimens (cf. Supplementary Figs. S6, S21) and can be related to a variety of amorphous Mn oxides^43^. One likely candidate in the case of the 632 cm^− 1^ peak of *D*. *armata* is todorokite, which is a mineral commonly found in Mn-rich hydrothermal deposits as well as in marine Mn nodules^44–46^. Todorokite is a secondary mineral product of more stable, crystalline Mn minerals such as birnessite, and forms as a transformation product at temperatures above 150 °C^47,48^. The distinct Raman band observed in *D*. *armata* bears similarity to reported spectra assigned to todorokite, in particular the strong band around the 630 cm^− 1^ position, with the MnO_6_ octahedron situated within the 600–610 cm^− 1^ spectral range^49^, with further diagnostic bands between 630 and 640 cm^− 1^^50^.

The distinctive colloform texture (Fig. 6) is frequently observed in hydrogenetic ferromanganese crusts^51–54^, suggesting a strong hydrogenetic component of the Myojin-sho Caldera *D*. *armata* encrustations. The texture of the coating of *D*. *armata*, with elongated depressions may also have a microbial component, as Fe and Mn oxides often form through microbial processes^55^. Todorokite is likewise associated with hydrothermalism, microbial activity and dynamic Mn redox cycling^56,57^. Although the formation mechanism of todorokite within *D*. *armata* coatings cannot be determined with certainty, it is likely that its occurrence is indicative of Mn oxide transformation under the influence of heated hydrothermal fluids derived from the immediate habitat of the gastropod. Although the coatings show features of several formation mechanisms (hydrogenetic, microbial), it cannot be determined with certainty which of these is mainly responsible for the precipitation of the coatings. It is likely that a combination of these leads to the distinct observable texture, as well as to the amorphous nature of the oxides.

The dual peaks with smaller shoulder peaks observed within *Desbruyeresia armata* coatings are further typical for varying forms of amorphous Mn oxides, which are efficient scavengers of bioessential and redox-sensitive trace metals^58^. Such metal inclusions within oxides can be detected using Raman spectroscopy. The dual peaks between 550 and 640 cm^− 1^ (Fig. 7d) are indicative of Mn oxides exhibiting inclusions of metal cations such as Ni, Cu, and Zn. Possible minerals exhibiting such inclusions are cryptomelane (K^+^), hollandite (Ba^2+^), coronadite (Pb^2+^), and romanechite (hydrated Ba^2+^), each exhibiting diagnostic Raman spectra^43^. A Mn oxide with similar peak intensity and position as seen in *D*. *armata* coatings is chalchophanite; a layered, hydrated Zn-bearing Mn oxide, yet this mineral is known for multiple characteristic peaks between 200 and 600 cm^− 1^ which are lacking in our samples^43^. The spectra shown in Fig. 7 bear some resemblance towards romanechite, due to the first prominent peak being higher than the second^59^. Romanechite is an amorphous, hydrated Ba-bearing Mn oxide known for its dual peaks around 570 and 643 cm^− 1^, as well as smaller shoulder peaks between 510 and 520 cm^− 1^^59,60^. The spectrum obtained in Fig. 7d shows dual peaks with opposite intensities to the other two, bearing similarities to hollandite^50^.

Positions of the Raman peaks for varying Mn oxide phases are also related to the Mn oxidation state. Mn^2+^O, Mn^3+^O, and Mn^4+^O have varying bond lengths between 2.25 Å (Mn^2+^) to 1.89Å (Mn^4+^). These differences in bonding lengths are reflected in the respective Raman spectra of Mn oxides within the coating, producing various peaks at specific intensities at 530 cm^− 1^ for Mn^2+,^ 570 cm^− 1^ for Mn^3+,^ and at ~ 630 cm^− 1^ for oxides containing Mn^4+^^43^. Mn oxides within the *Desbruyeresia armata* coatings likely contain Mn in varying redox states from Mn^2+^ to Mn^4+^, derived largely from amorphous hollandite and/or birnessite group minerals. Taken together, the variety of Raman spectra collected from *D*. *armata* suggests a series of poorly crystalline, amorphous, partially hydrated, and likely metastable Mn oxides incorporating Ba, Zn, Pb, and possibly Cu to different degrees, corresponding to results obtained from SEM mapping. Interestingly, despite element maps that suggest the presence of Fe-bearing minerals within the coatings alongside Mn oxides, no concrete mineralogical evidence for Fe oxides or any other Fe minerals could be detected in the Raman spectra. This can be due to the overall non-crystalline and highly heterogeneous nature of the coatings, composed of a mixture of various amorphous phases that may mask spectra that can be identified as Fe oxides. Two Raman spectra from *D. marisindica* and *A. marisindica* from Kairei vent field show peak positions at ~ 1350 and 1600 cm^− 1^ (Supplementary Figs. S21, S26), which identify the D-bands and G-bands of various types of organic matter^61,62^. Increasing peak sharpness has been previously interpreted as an indicator of diagenetic alteration of carbon compounds during pressure and temperature increase^63^, yet the diffusive nature of the peaks obtained for the gastropod specimens suggests fresh, undegraded organic matter within the shell coatings.

Mn and Fe oxides and oxyhydroxides have the ability to scavenge some elements from the seawater, such as Co, Ni, and P. Co and Ni are usually more enriched in hydrogenetic than hydrothermal deposits^42^, but they can also be incorporated into hydrothermal sulfides or scavenged by particles present in hydrothermal plumes^64^. Small amounts of Co and Ni, as well as large amounts of P were present in the *Desbruyeresia armata* coating, which is also suggested by Raman data (Fig. 7). Apart from Mn and Fe oxides, the coating was rich in S and Pb, as well as Zn and Cu (Supplementary Figs S14, Fig. S16). Galena and sphalerite are indeed said to be the dominant sulfide phases at the Myojin-sho Caldera^28^. Pyrite and chalcopyrite are also commonly recovered from this site^28^. Furthermore, volcanogenic massive sulfides are frequently enriched in Zn, Pb, and Cu^65^. The presence of abundant Pb, Zn, and Cu in the coatings of *D*. *armata* is also consistent with the substrate of Myojin-sho Caldera, which consists of andesite, rhyolite, and dacite (InterRidge Vents Database v. 3.4^11^;. Moreover, vent fluids at volcanic arc settings are often enriched in Pb^66^.

### *Desbruyeresia marisindica* and *Alviniconcha marisindica*, Kairei vent field, Central Indian Ridge

Both *Desbruyeresia marisindica* and *Alviniconcha marisindica* collected from the Kairei vent field are covered with coatings containing abundant Fe (Figs. 8, 9 and 10; Supplementary Figs S18, S19, S20). However, in *A. marisindica* Fe occurs most likely in the form of Fe sulfides, while in case of *D. marisindica* it is Fe oxides. High Fe abundance is consistent with relatively high Fe/Mn ratios reported from Kairei^29^. Furthermore, the EDS elemental maps suggest a presence of Fe-Cu sulfides on the surface of both studied gastropods (Supplementary Figs, S18, S23, S24), however they are more abundant in the coating of *A. marisindica*. This specimen comes from the hottest part of an active chimney, where Cu-rich sulfides are especially common in the proximity of the vent orifice since they need very high temperatures to form^67^. Although the distribution of Fe and S is very similar to *A. marisindica* from the Edmond vent field, Raman data collected from Kairei coatings cannot determine the presence of pyrite with the same degree of confidence. The spectra show several erratic small peaks between 150 and 550 cm^− 1^, yet cannot be assigned to a particular Fe-bearing mineral with certainty (Supplementary Fig. S25).

Chalcopyrite, sphalerite, and pyrite are dominant massive sulfides present at Kairei and mostly enriched in Co (up to 1320 ppm, average 401 ppm; average Co concentration in mid-ocean ridge basalts: 44 ppm^30,68^; Co is present on shell surfaces of *D. marisindica* and *A. marisindica* (Supplementary Figs S23, S24) in trace amounts and following the distribution of larger sulfide deposits. The Co enrichment is commonly associated with ultramafic-hosted hydrothermal systems^9,11^ and the Kairei vent field is generally thought to be such^30,31^. This suggests that Fe sulfides such as pyrite may be present within the coatings, yet cannot be identified mineralogically due to the amorphous and heterogeneous character of the coatings. The abundance of Ca in the mineral coating is consistent with high abundance of Ca and relatively high Ca/Cl ratio in the Kairei vent fluids. Although this is usually interpreted as evidence of the albitization of the host rock (i.e. a transformation of Ca and K feldspar into albite), in case of the Kairei vent field it is thought to be a result of the decomposition of clinopyroxene^31,69^. High abundance of P and Si, visible both on the surface and on the cross section images of *D. marisindica* (Fig. 9; Supplementary Fig. S20), may suggest the presence of Fe silicates and Fe oxides in the coating. In fact, Fe and Si are the most abundant elements on the cross-section images of the coating. The enrichment of Fe and SiO_2_ has been linked to the activity of Fe-oxidizing bacteria at the Edmond vent field^32^, however, there is no morphological evidence for microbial activity in the coating of *D. marisindica* specimen from Kairei vent field studied herein.

### *Alviniconcha marisindica*, Edmond vent field, Central Indian Ridge

The coating of *A. marisindica* from the Edmond vent field consists mainly of Fe, S, and Si (Supplementary Fig. S28). Hydrothermal fluids from Edmond are reported to have relatively high Fe content—ca. two times higher than at Kairei^30^. Although Fe and S co-occur locally, their patterns of distribution are different. Furthermore, the co-occurrence of Fe and P, as well as the reddish color of the coating suggest Fe oxides and oxyhydroxides are dominant. This is consistent with the fact that massive sulfides recovered from the Edmond vent field are typically coated by reddish-brown ferric oxides^33^. Moreover, the accumulation of orange Fe oxyhydroxide sediments, hosting microbial mats, is a characteristic feature of the locality^29^. The co-occurrence of Fe and Si may also be explained by the activity of Fe-oxidizing bacteria, which has been recognized at Edmond^32^.

Raman spectra collected from coatings of *A. marisindica* confirm the presence of Fe sulfides within areas of overlapping Fe and S content (Fig. 12). As opposed to spectra obtained from *A. marisindica* from the Kairei Vent field, two distinct peaks at 338 and 376 cm^− 1^ are almost identical to ideal pyrite (cf. RRUFF database, https://rruff.info/), thus confirming the presence of pyrite within the coating. This spectrum is similar to that obtained for *A. marisindica* from the Kairei vent field (cf. Supplementary Fig. S25). The Fe and Fe-Cu sulfides present in the coating of *A. marisindica* from Edmond vent field are significantly enriched in Bi (or Pb) (Supplementary Figs S27, S29), which is usually associated with significant sedimentary component in the hydrothermal system^39^. High Ca abundance in the coating of Edmond vent field specimen may be explained by Ca contents and Ca/Cl ratio in fluids, which may be a result of the albitization of host rock^29,31^. Ba is also enriched in the emitted fluids^31^ and is present in the coating of *A. marisindica*.

### Biological significance of the coatings

There is no evidence for mineral replacement of the studied vent gastropod shells. Coatings on the *L. nux* (Fig. 3c, d) and *A. marisindica* (Fig. 11a, b) specimens are separated from the shell by a relatively thick layer of periostracum. In case of *Desbruyeresia armata* (Fig. 5e), the coating is partly overgrown by the shell of the animal, which shows that its formation is contemporary with the shell growth.

The presence of often relatively thick coatings on the shell surface may impact the host animal. Depending on its thickness the shell may become too heavy and difficult for the gastropod to freely move around. However, all specimens studied here were collected alive, including *Desbruyeresia armata* from Myojin-sho Caldera with the thickest coating, suggesting that heavy shell encrustation may not necessarily be problematic for the animal. Moreover, the coating most likely provides the snail with additional protection against corrosive hydrothermal fluids. None of the specimens studied here have visible signs of shell dissolution, whereas other vent gastropods are commonly found with missing protoconchs and dissolution pits in the teleoconch^23^. Rapid coating formation on the shell surface may protect gastropods from early dissolution of the teleoconch. Since the composition of encrustations of studied specimens changes in relation to the composition of vent fluids and with distance to the active venting sites, it is likely not a biological adaptation to life in extreme environment but rather a passive, environmental process of encrustation of the shell surface. While microorganisms may play a role in coating formation, species studied likely do not mediate the process. This is in contrast to the scaly-foot snail *Chrysomallon squamiferum*, which removes S likely originating from its endosymbionts via special channels in its scales; the S then becomes the basis for forming iron sulfide when the iron concentration is high in the environment^70^.

By protecting the shell from early dissolution, these coatings have taphonomic relevance as they may increase chances of successful fossilization of gastropod shells. Fossilized vent gastropods are known from only three localities^4^: from Upper Cretaceous Troodos Ophiolite in Cyprus^71,72^, Lower Jurassic Figueroa deposit in California^73^, and Yaman Kasy Deposit in Ural Mountains, Russia^74^. All these fossils are preserved as thin external molds of pyrite. The original shell material is always absent, however, ornamentation or periostracum details are usually preserved. In contrast, coatings of gastropod specimens studied here exhibit much more diverse compositions (Fe, Cu and Zn sulfides, Fe and Mn oxides, Ca and Ba sulfates).

Extremely rapid mineralization of animal tissues occurs in close proximity to active venting and has been reported to take less than a year for the tubes of alvinellid Pompeii worms^75^. Since hydrothermal fluids are usually acidic, the dissolution of carbonate shells happens rapidly and is faster (a few years) in areas closer to the active venting^76^. Therefore, rapid mineralization in hotter parts of the chimney is probably a prerequisite for subsequent preservation of vent fauna in the fossil record^72^. Out of six specimens studied here, two were collected from the hottest part of the chimney (*L. nux* from Iheya North vent field, Okinawa Trough, and *A. marisindica* from Kairei vent field, Central Indian Ridge). *Desbruyeresia marisindica* was recovered from the active chimney wall, but not the hottest area. The remaining three specimens were associated with low-temperature diffuse flows. While Fe (and some Cu) sulfides indeed dominate the coatings of *A. marisindica* from Kairei, the shell surface of *L. nux* from Iheya North is rich in Ca sulfates and Pb sulfides. However, Kairei and Iheya North differ substantially in their geochemical conditions.

While coatings may not be the first step of fossilization process, they may protect gastropod shells from corrosive hydrothermal fluid, delaying their dissolution and making them more likely to be incorporated into the fossil record.

## Conclusions

Coatings of six gastropod specimens from four different hydrothermal vent fields were examined. Their chemical composition changed in relation to the composition of vent fluids and to the distance to the vent orifice. The most commonly observed minerals are Fe and Mn oxides, pyrite, galena, and sphalerite. No conclusive evidence confirming the role of microorganisms in the formation of coatings was found, however, the texture of that on the *Desbruyeresia armata* shell suggest it may be the case for this specimen. Nevertheless, the species studied here likely have no biological control over coating formation. The examination of gastropod shells cross-sections indicated no replacement of the shell. The coating is partially overgrown by the shell in one example, suggesting the shell growth can be contemporary with coating formation. Although encrustations may negatively impact the movement of the snail, they provide protection from corrosive hydrothermal fluids (and possibly predation), which may also increase the chances of fossilization after the animal’s death. While fossils of ancient gastropods are all thin external molds of pyrite, modern shell encrustations have much more diverse composition. Still, pyrite is a dominant mineral phase in coatings of specimens collected from areas of high-temperature active venting. Known gastropod fossils were probably preserved in similar high-temperature conditions, which may partially explain their scarce fossil record. Even though coatings do not appear to always be the first step of the fossilization, they may play a crucial role in delaying the dissolution of the gastropod shell, as all six specimens studied exhibited no visible damage to the calcareous shell.

## Supplementary Information

Below is the link to the electronic supplementary material.

Supplementary Material 1

## Data Availability

Raw data for this study is available at OSF: [10.17605/OSF.IO/V7PCQ].
