# Supplementary material for: Diversity of coatings on hydrothermal vent gastropods
Source: Sci Rep. 2025 Dec 2;15:45807. doi: 10.1038/s41598-025-29638-3 (PMC12756243; doi:10.1038/s41598-025-29638-3)
Supplement: Supplementary file 1 — Supplementary Material 1 [file 41598_2025_29638_MOESM1_ESM.docx]

**Diversity of mineral coatings on hydrothermal vent gastropods**

**⃰Agata Bonk^1^, Krzysztof Hryniewicz^1^, Paweł Bącal^1^, Daniel Smrzka^2,3^, Chong Chen^4^, Crispin T.S. Little^5^**

1. Institute of Paleobiology, Polish Academy of Sciences, ul. Twarda 51/55, 00-818 Warszawa, Poland.
2. MARUM, Zentrum für Marine Umweltwissenschaften, 28359 Bremen, Germany.
3. 3 Fachbereich Geowissenschaften, Universität Bremen, 28359 Bremen, Germany
4. X-STAR, Japan Agency for Marine-Earth Science and Technology (JAMSTEC), 2-15 Natsushima-cho, Yokosuka 237-0061, Japan.
5. School of Earth and Environment, University of Leeds, Woodhouse Lane, Leeds LS2 9JT, UK.

⃰Agata Bonk: abonk@twarda.pan.pl

ORCID: AB, 0000-0002-0664-5577; KH, 0000-0002-0664-5577; PB, 0000-0001-7935-639X; DS, 0000-0001-8437-3039; CC, 0000-0002-5035-4021; CTSL, 0000-0002-1917-4460

**SUPPLEMENTARY MATERIAL**

**A**

**D**

**C**

**B**

**
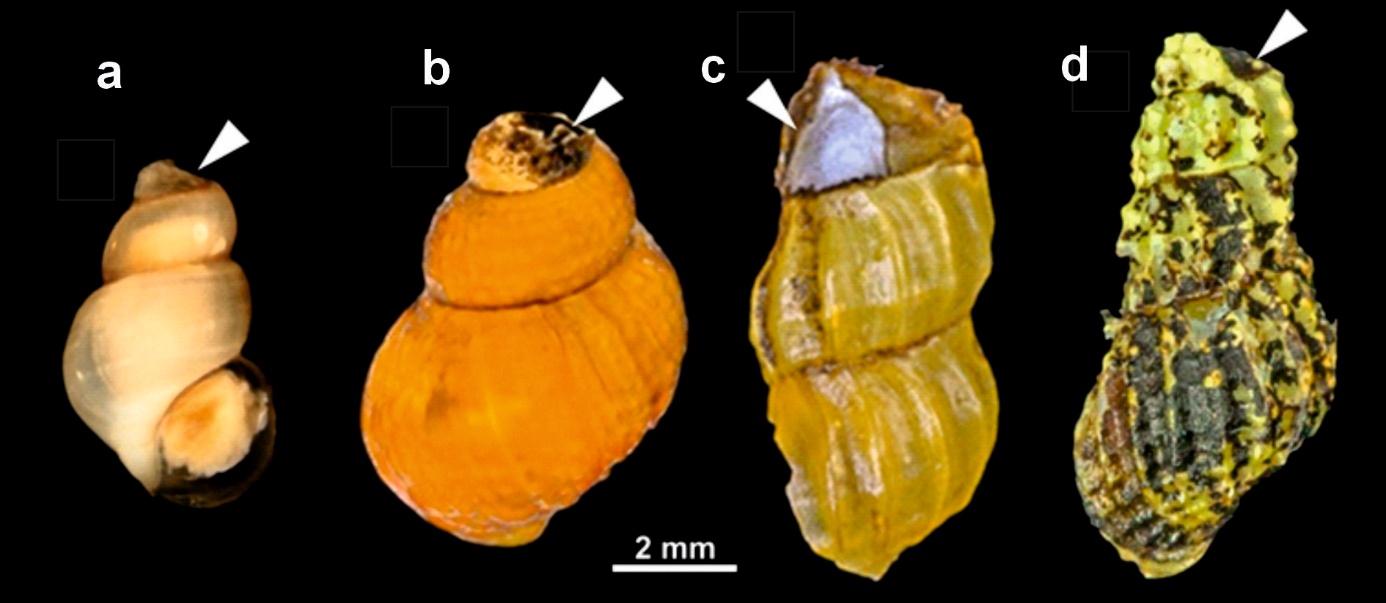
Figure S1.** Examples of vent gastropods affected by dissolution; (a) *Provanna lucida*, Minami-Ensei Knoll vent site, Okinawa Trough (after Sasaki et al., 2016, fig. 6a) (b) *Provanna beebei* (Linse et al., 2019) Anemone Field, Beebe Vent Field, Mid-Cayman Spreading Centre, Caribbean Sea (after Linse et al. 2019, fig. 2j) (c) *Desbruyeresia costata* (Chen et al., 2019) Izena Hole hydrothermal vent field, Okinawa Trough (d) *Desbruyeresia armata* Bayonesse Knoll hydrothermal vent site, Izu-Ogasawara Arc (after Chen et al. 2019, fig. 2f). Arrowheads indicate area where protoconch was attached prior to detachment.


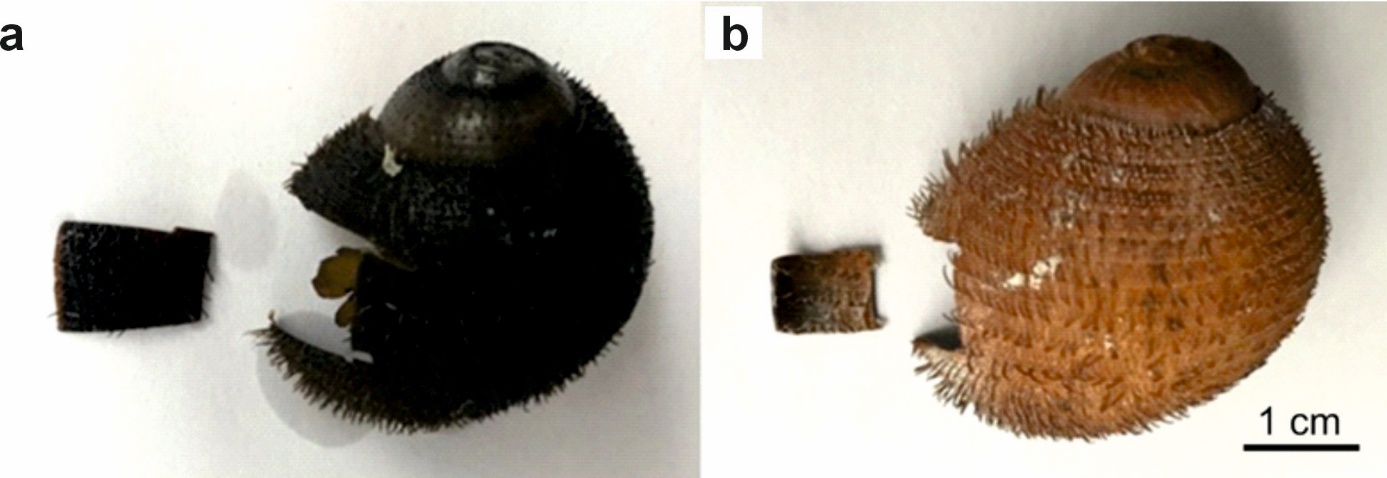


**Figure S2.** A shell cut out from specimens of *Alviniconcha marisindica* from (a) the Kairei Vent Field, ZPAL Ga.22/5, and (b) the Edmond Vent Field, ZPAL Ga.22/6. Scale bar applies to both (a) and (b).

**
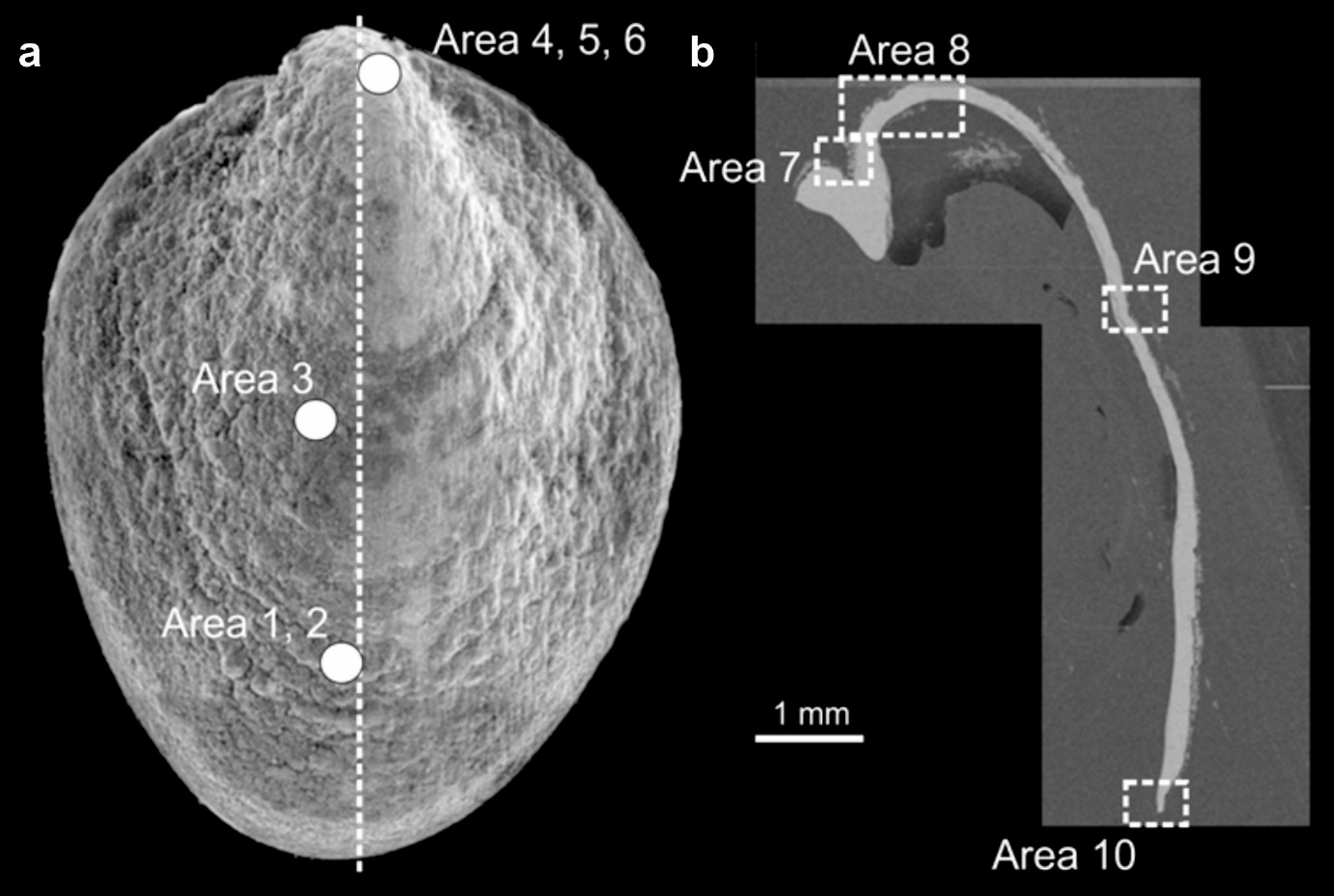
**

**Figure S3.** *Lepetodrilus nux*, ZPAL Ga.22/1, Original Site, Iheya North Vent Field, Okinawa Trough, Pacific Ocean; (a) SEM surface view; dashed line indicates the cross-section plane; (b) SEM BSE view of the cross-section. Areas where elemental maps were created on the shell surface and cross-section are indicated.


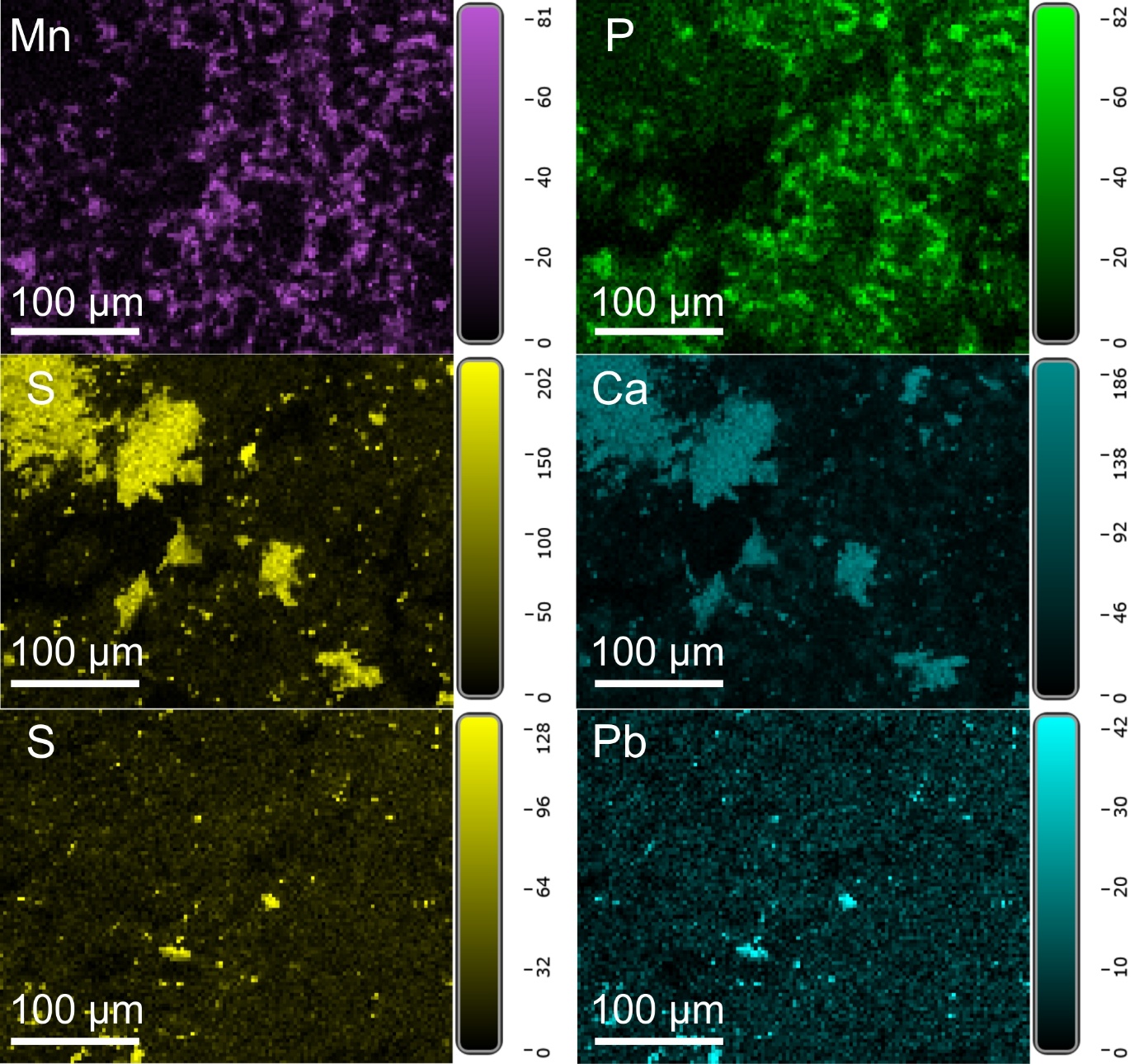


**Figure S4.** EDS elemental maps of the selected areas on the shell surface of *Lepetodrilus nux*, ZPAL Ga.22/1, Iheya North Vent Field, Okinawa Trough, Pacific Ocean. Top two rows in Area 10, bottom rown in Area 7 (See Fig. S3).


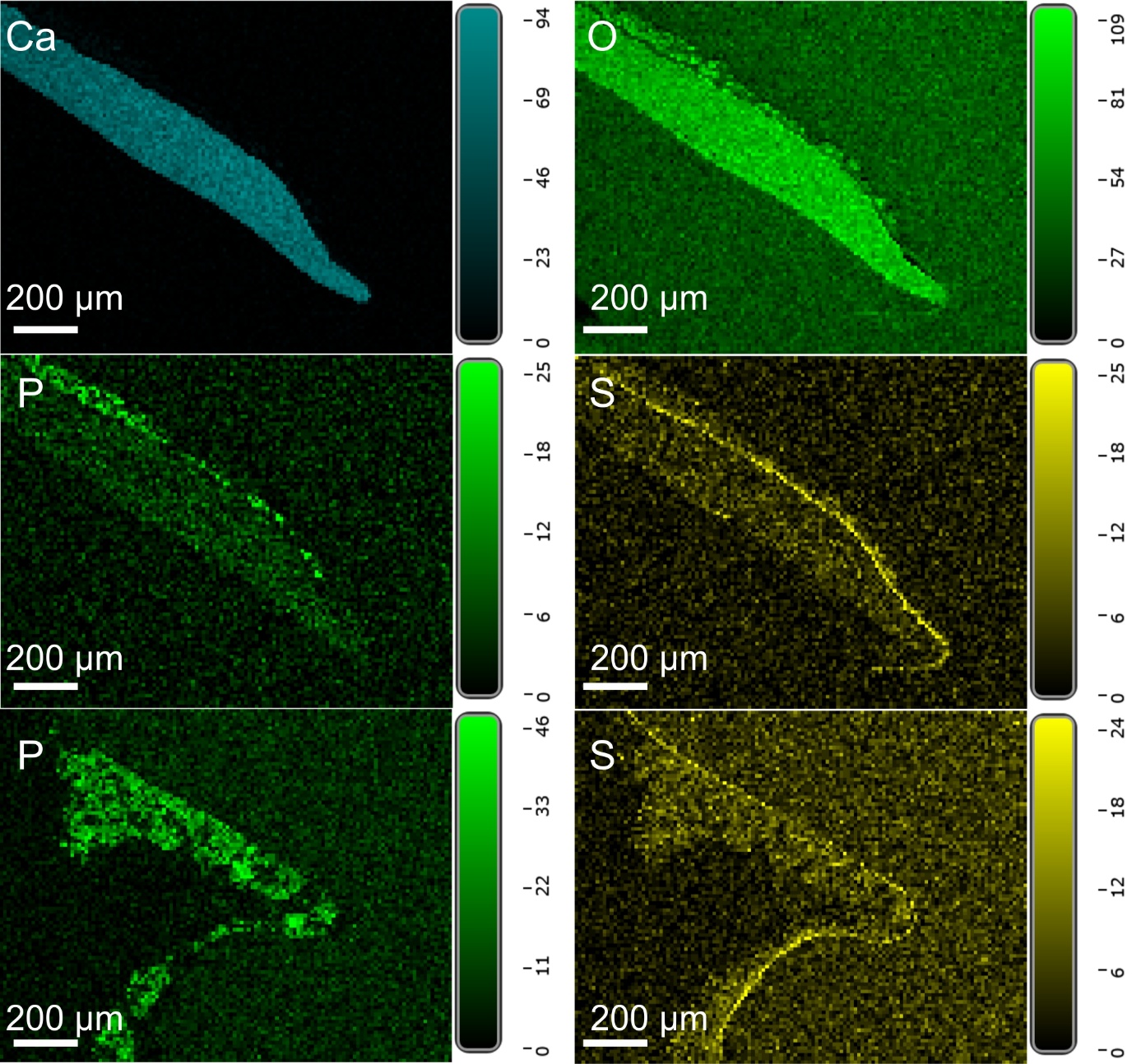


**Figure S5.** EDS elemental maps of the cross-section through the shell of *Lepetodrilus nux*. ZPAL Ga.22/1. They show distribution and abundance of Ca, O, P and S. Top and middle row in Area 10; lower row in Area 7 (see Fig. S3).


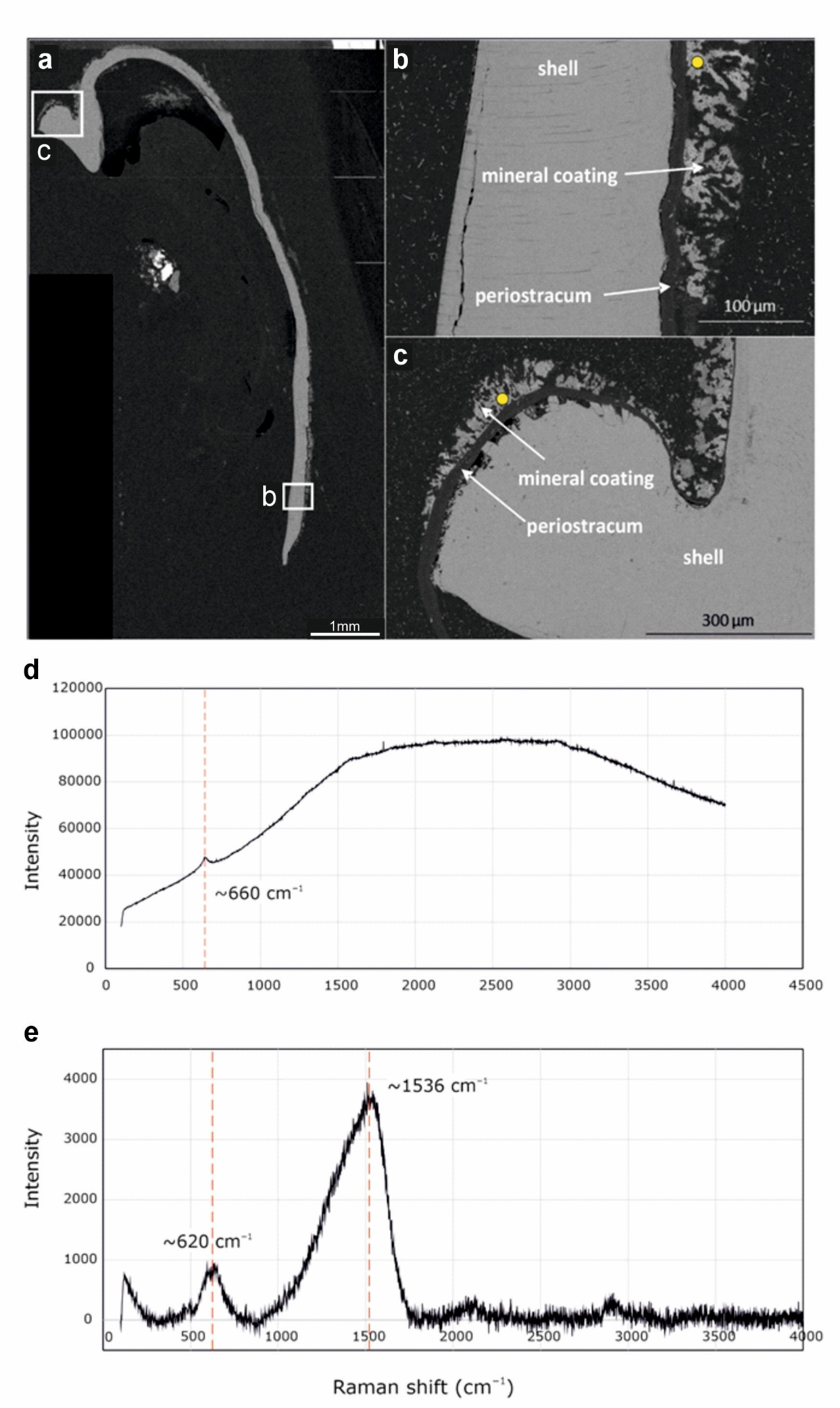


**Figure S6.** Raman spectrum of mineral coating from *L*. *nux*, ZPAL Ga.22/1, Iheya North Vent Field (a) SEM overview image of *L*. *nux* cross-section, highlighting the two areas where Raman measurements were conducted (marked by yellow dots). Lettered insets in (a) correspond to images (b) and (c). (d) Raman spectrum of image (b), showing a diffuse hump at around 660 cm^−1^. (e): Raman spectrum of image (c), showing a two peaks at 620 and 1536 cm^−1^.


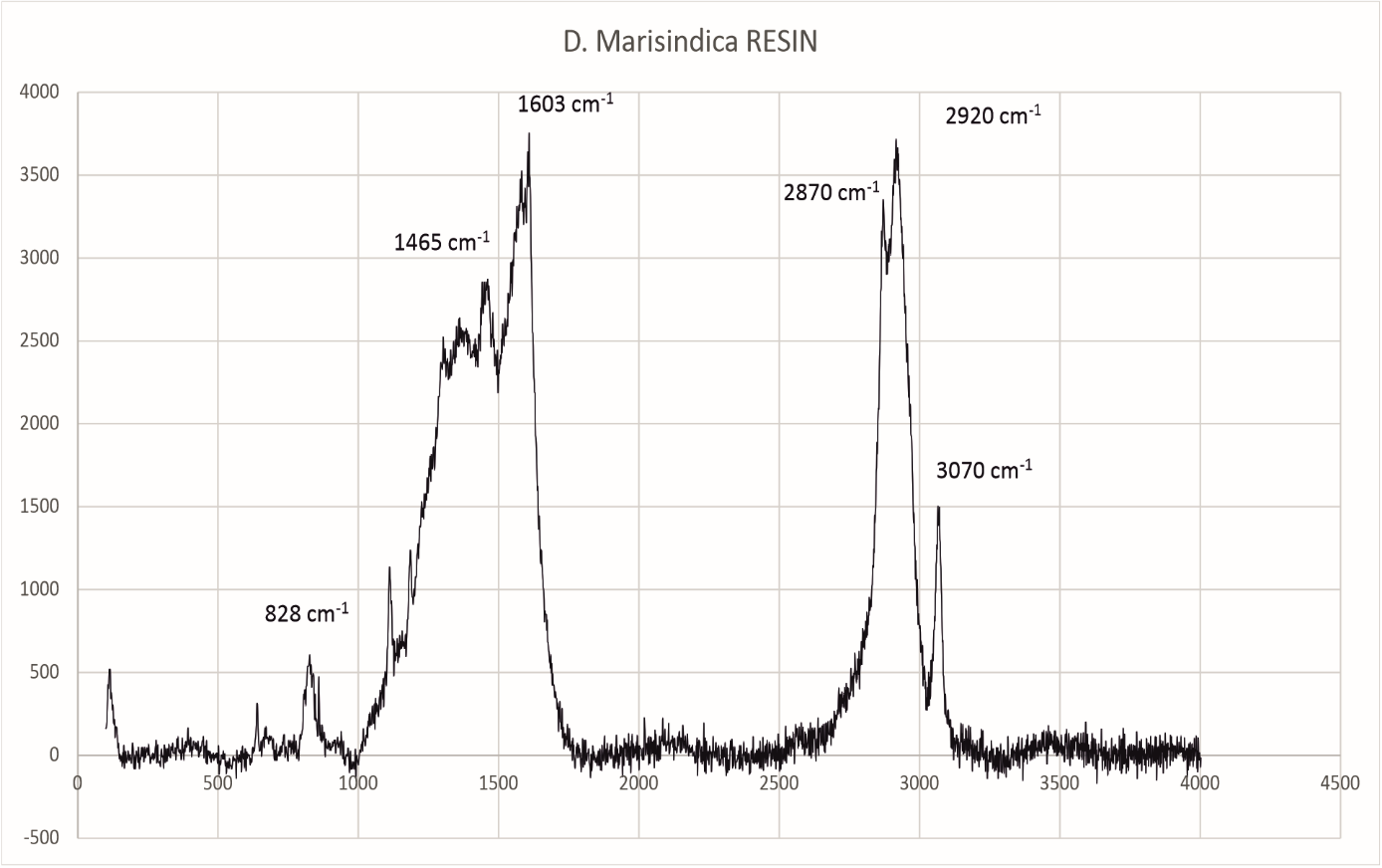


**Figure S7.** Raman spectrum from resin material of the *D*. *marisindica*, ZPAL Ga.22/4, Kairei Vent Field, sample, showing several distinct peaks.


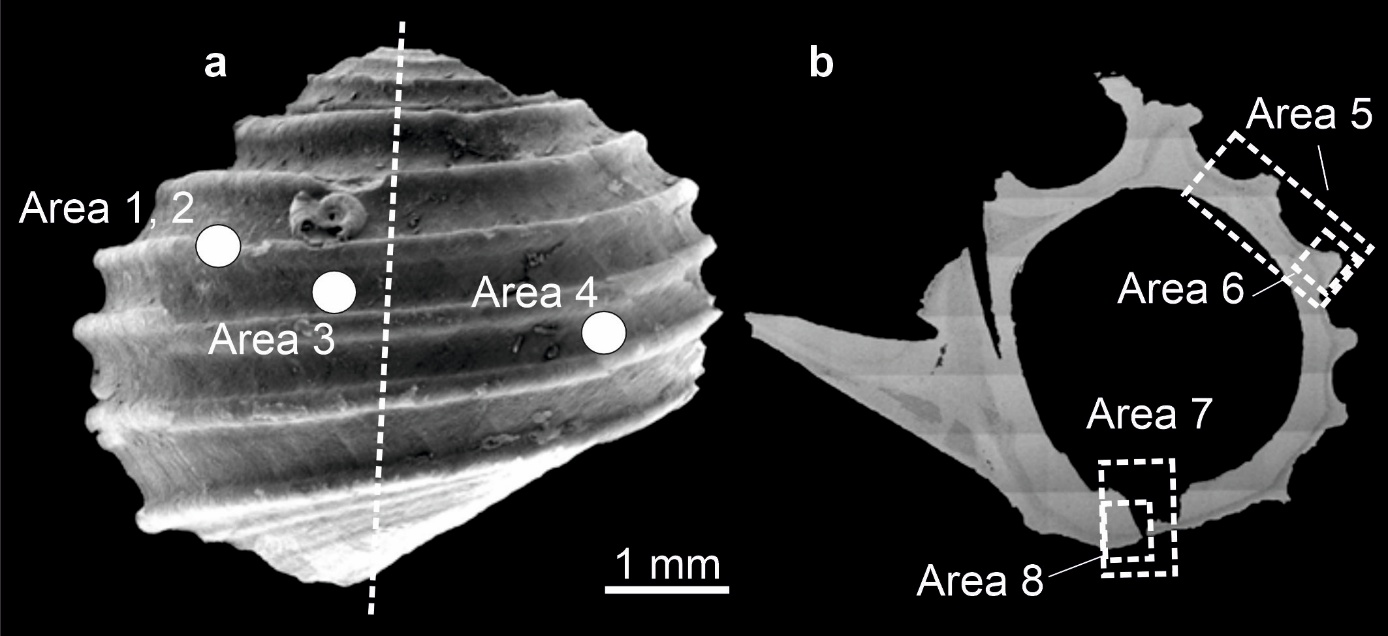


**Figure S8.** *Cantrainea jamsteci*, ZPAL Ga.22/2, Aki Site, Iheya North Vent Field, Okinawa Trough; (a) SEM surface view; dashed line indicates the cross-section plane; (b) SEM BSE view of the cross-section. Areas where elemental maps were created on the shell surface and cross-section are indicated.


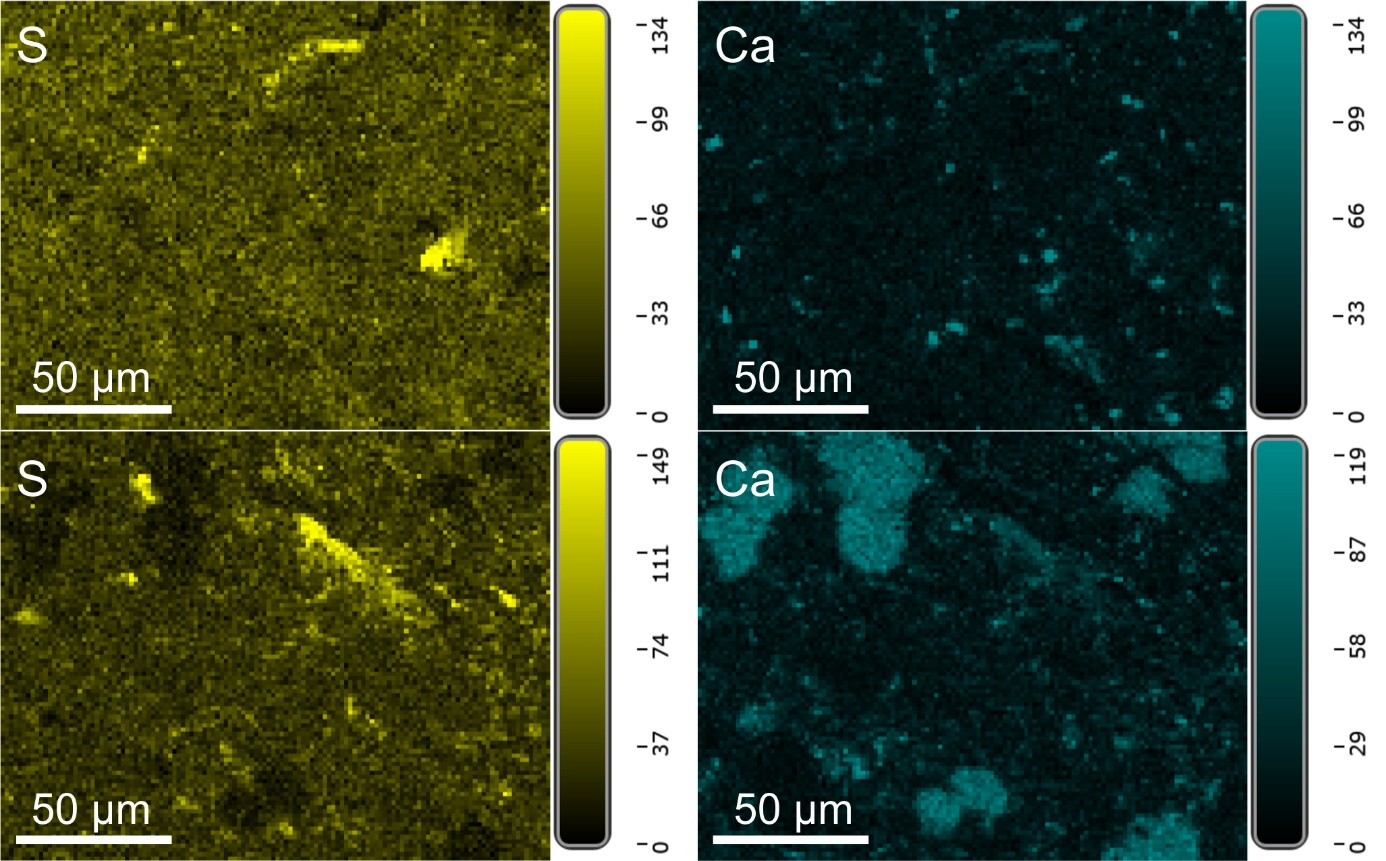


**S**

**Figure S9.** EDS elemental maps of the shell surface of *Cantrainea jamsteci*, ZPAL Ga.22/2, Aki Site, Iheya North Vent Field, Okinawa Trough. Top row in Area 4; lower row in Area 3 (see Fig. S8).


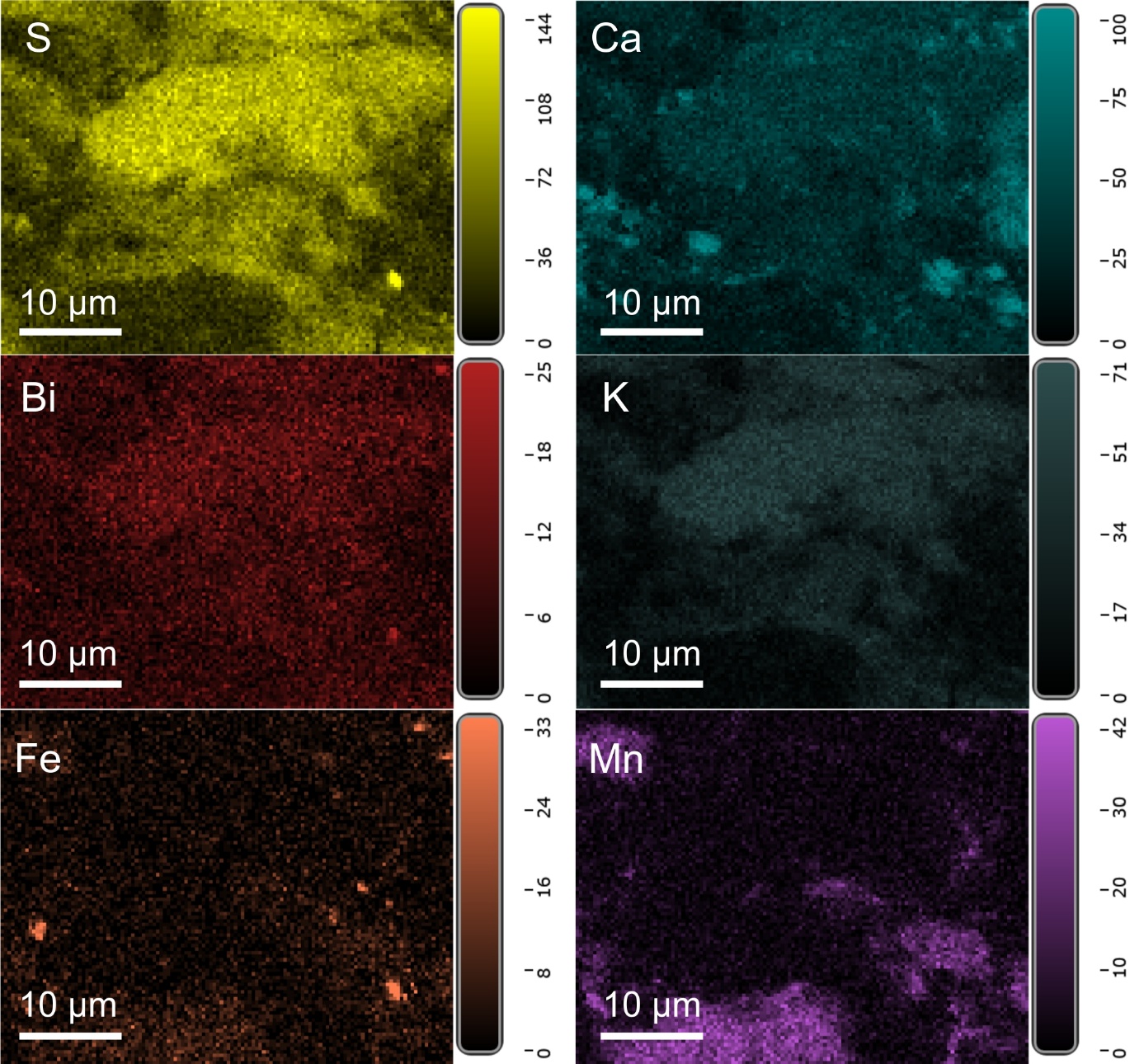


**Figure S10.** EDS elemental maps of S, Ca, Bi, K, Fe and Mn on the shell surface of *Cantrainea jamsteci*, ZPAL Ga.22/2, Aki Site, Iheya North Vent Field, Okinawa Trough. Area 2 (see Fig. S8).


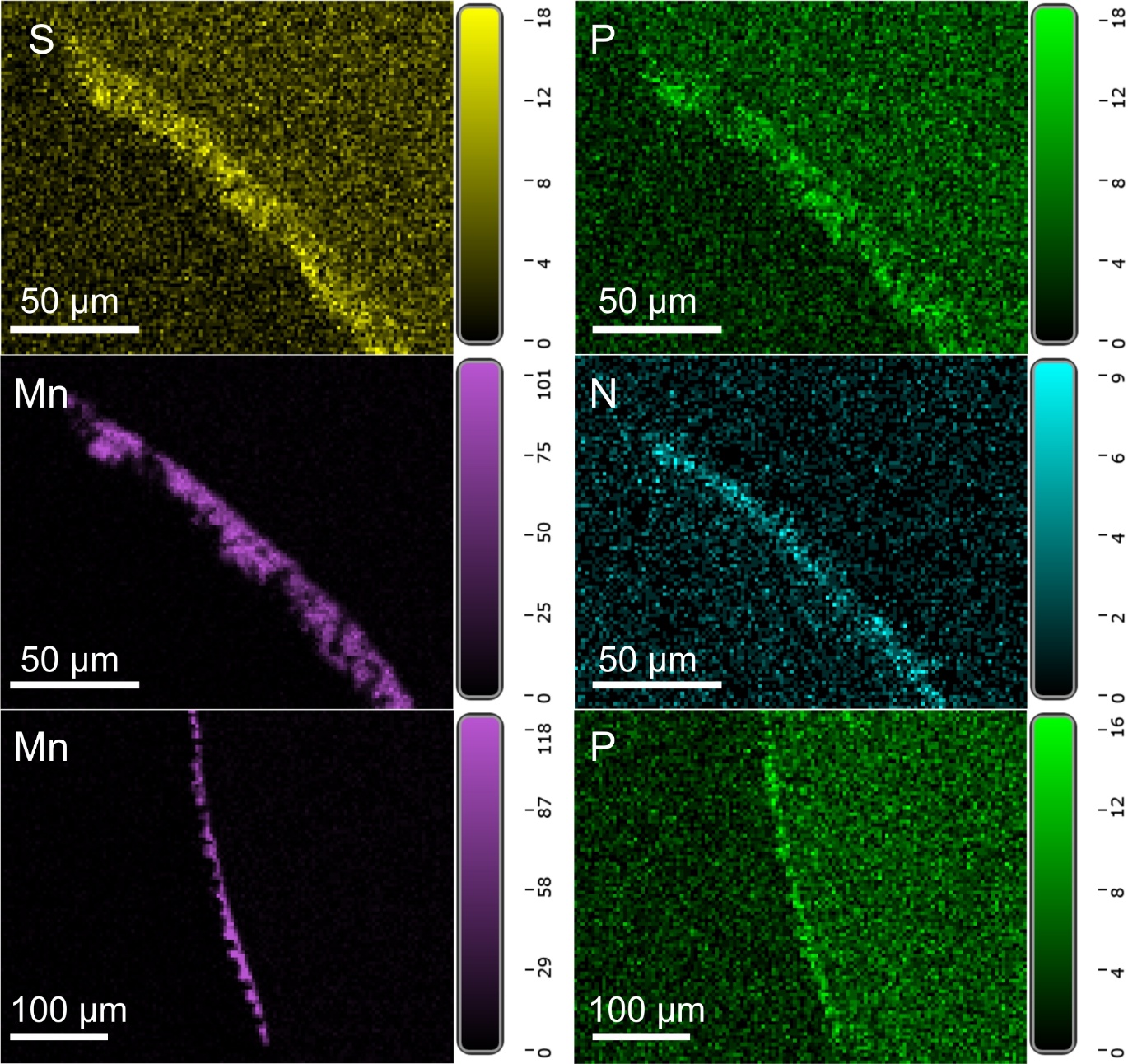


**Figure S11.** EDS elemental maps of the cross-section through the shell of *Cantrainea jamsteci*, ZPAL Ga.22/2, Aki Site, Iheya North Vent Field, Okinawa Trough, Pacific Ocean. Top and middle row in Area 6; bottom row in Area 8 (see Fig. S8)


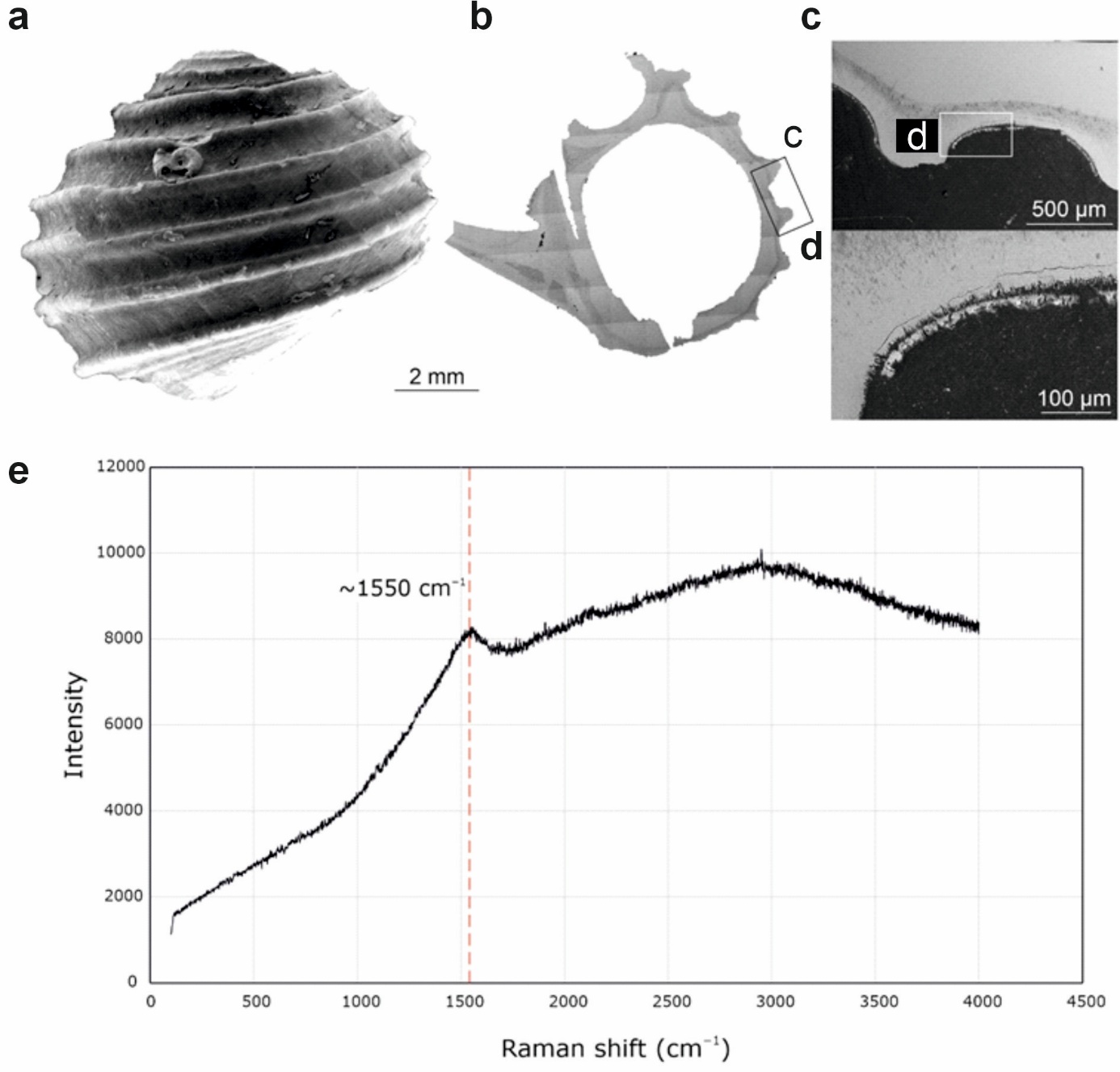


**Figure S12.** Raman spectrum of mineral coating from *C. jamsteci*, ZPAL Ga.22/2, Aki Site, Iheya North Vent Field, Okinawa Trough, with a diffusive, broad peak at ~1550 cm^−1^. (a) SEM overview image of *C*. *jamsteci*, (b) cross-section. Lettered insets in (b) and (c) correspond to images (c) and (d). (e) Raman spectrum of *C*. *jamsteci*.


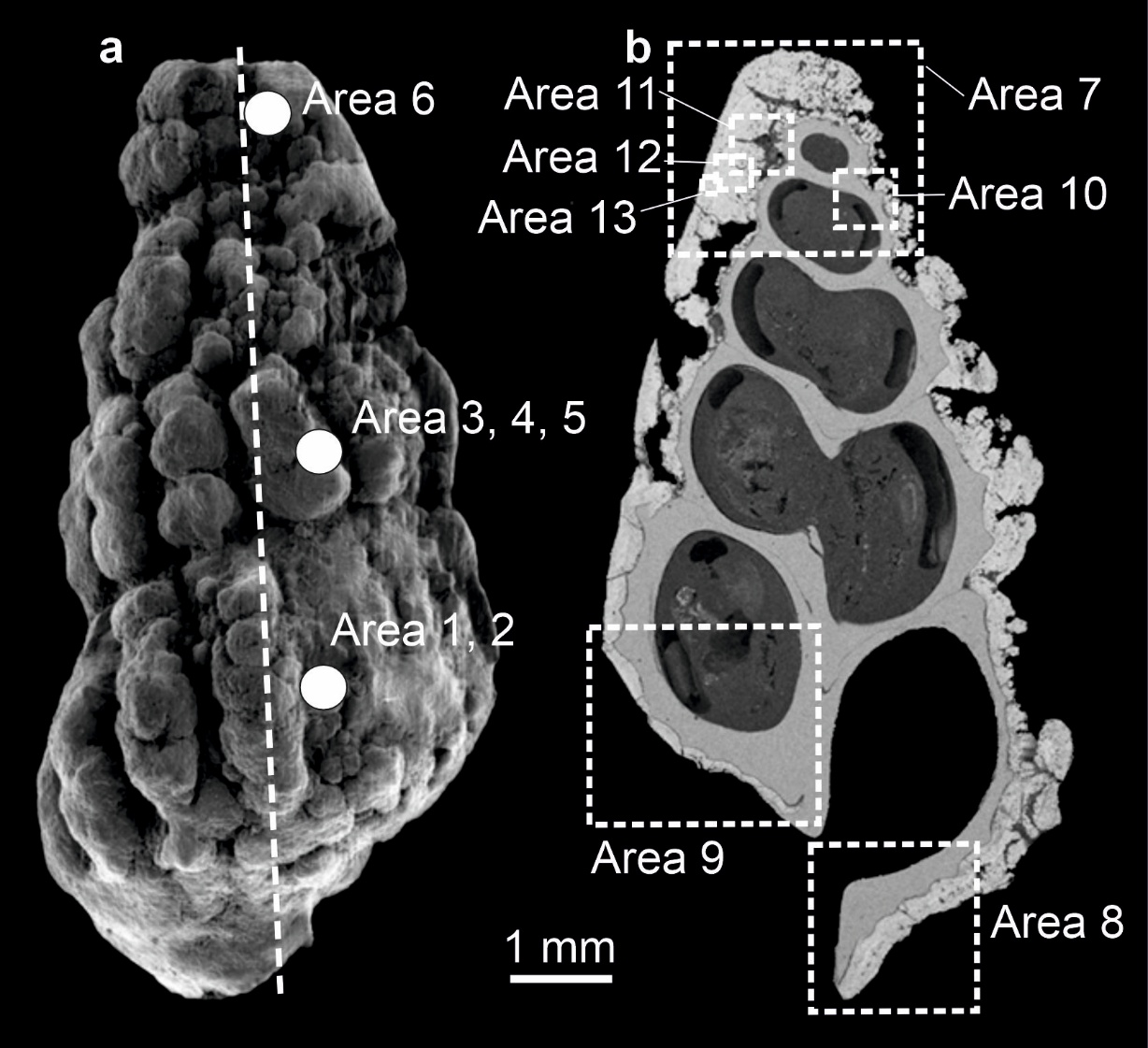


**Figure S13.** *Desbruyeresia armata*, ZPAL Ga.22/3, Myojin-sho Caldera, Izu-Ogasawara arc, Pacific Ocean; (a) SEM surface view; dashed line indicates the cross-section plane (b) SEM BSE view of cross-section. Areas where elemental maps were created on the shell surface and cross-section are indicated.


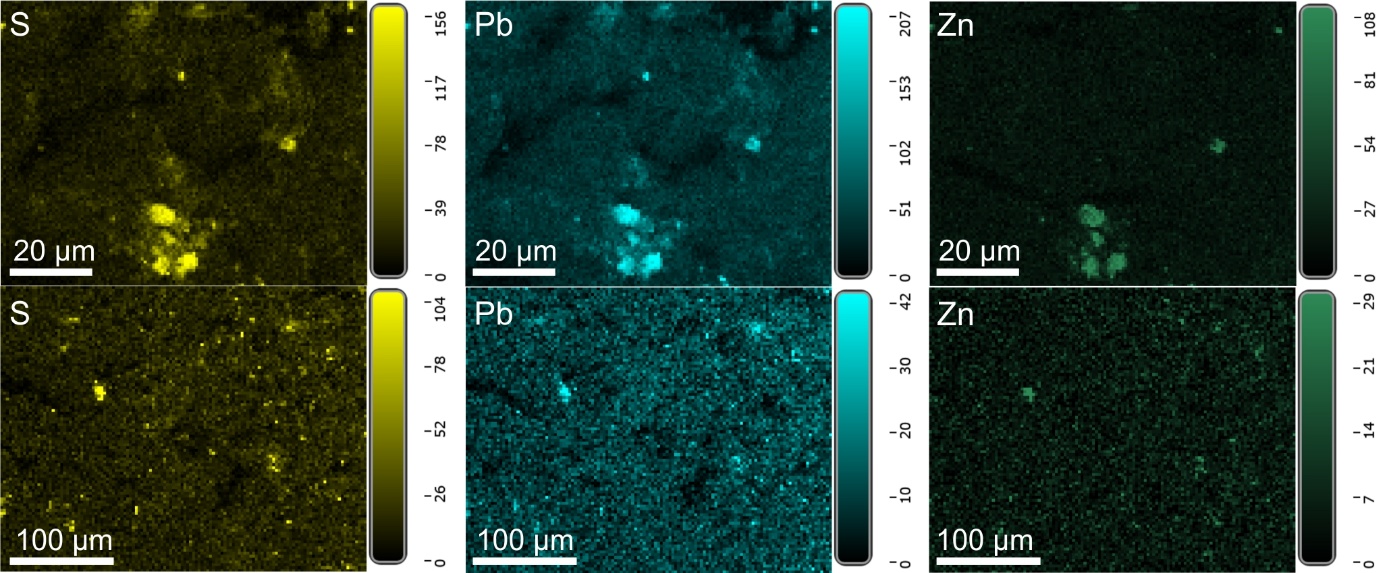


**Figure S14.** EDS elemental maps of the shell surface of *Desbruyeresia armata*, ZPAL Ga.22/3, Myojin-sho Caldera, Izu-Ogasawara arc, Pacific Ocean. Top row in Area 5, bottom row in Area 3 (see Fig. S13).


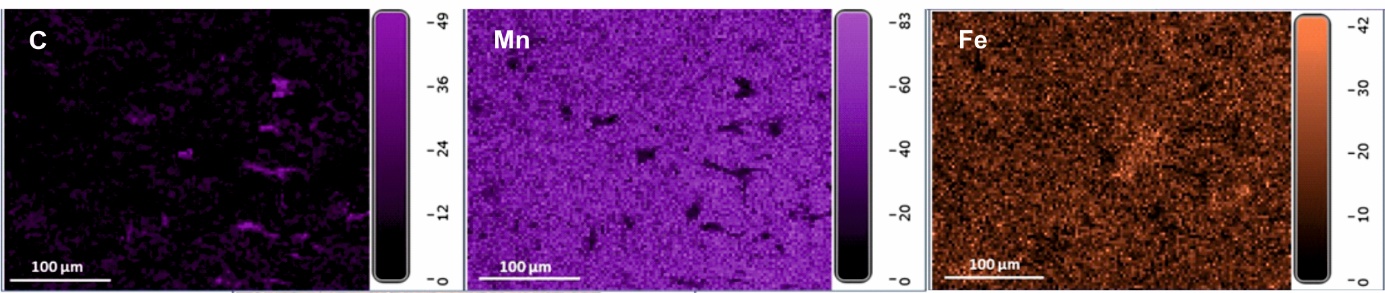


**Figure S15.** EDS elemental maps of the shell surface of *Desbruyeresia armata*, ZPAL Ga.22/3, Myojin-sho Caldera, Izu-Ogasawara arc, Pacific Ocean. Area 3 (See Fig. S13)


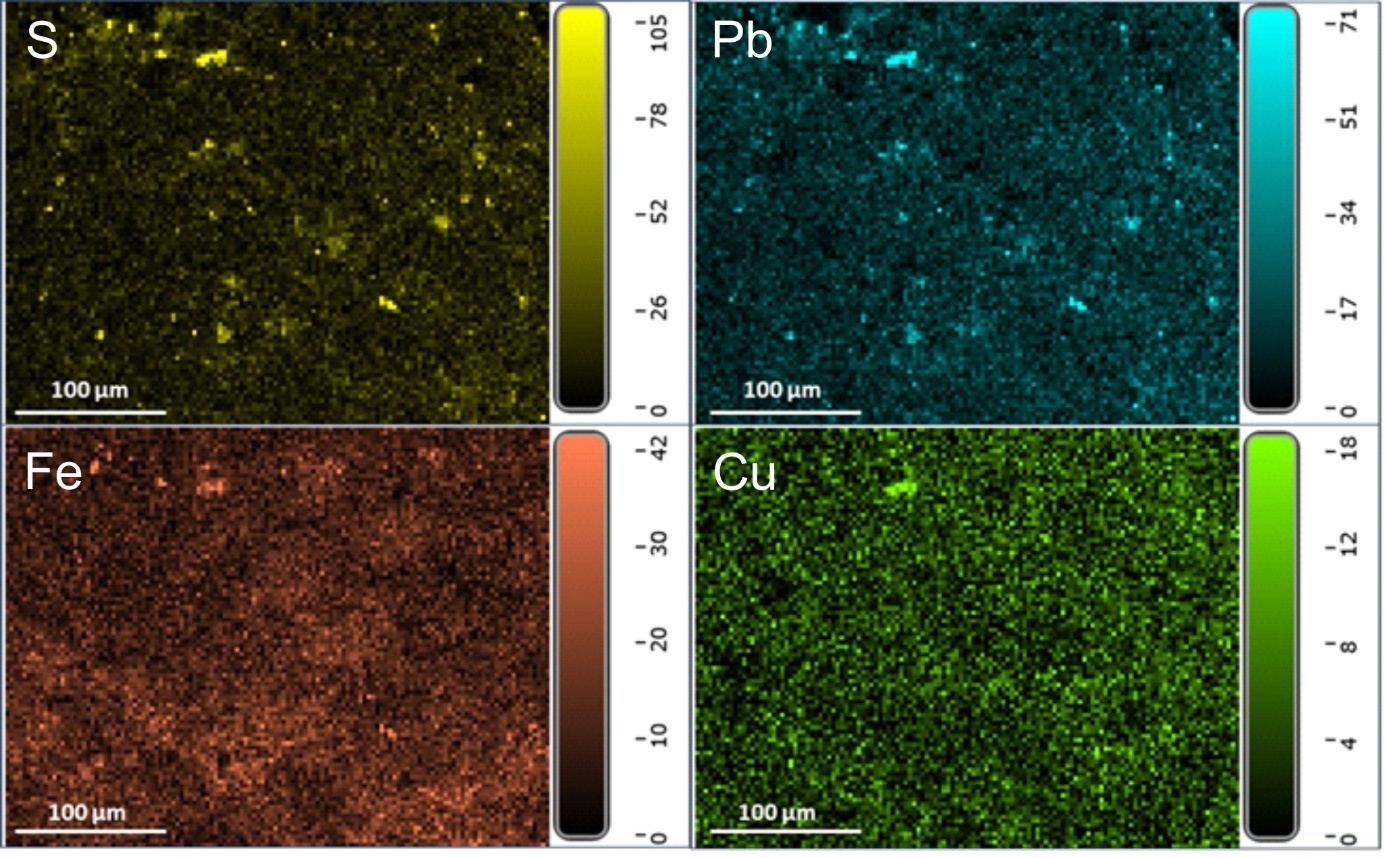


**Figure S16.** EDS elemental maps of Area 2 of the shell surface of *Desbruyeresia armata*, ZPAL Ga.22/3, Myojin-sho Caldera, Izu-Ogasawara arc, Pacific Ocean. Area 2 (see Fig. S13).


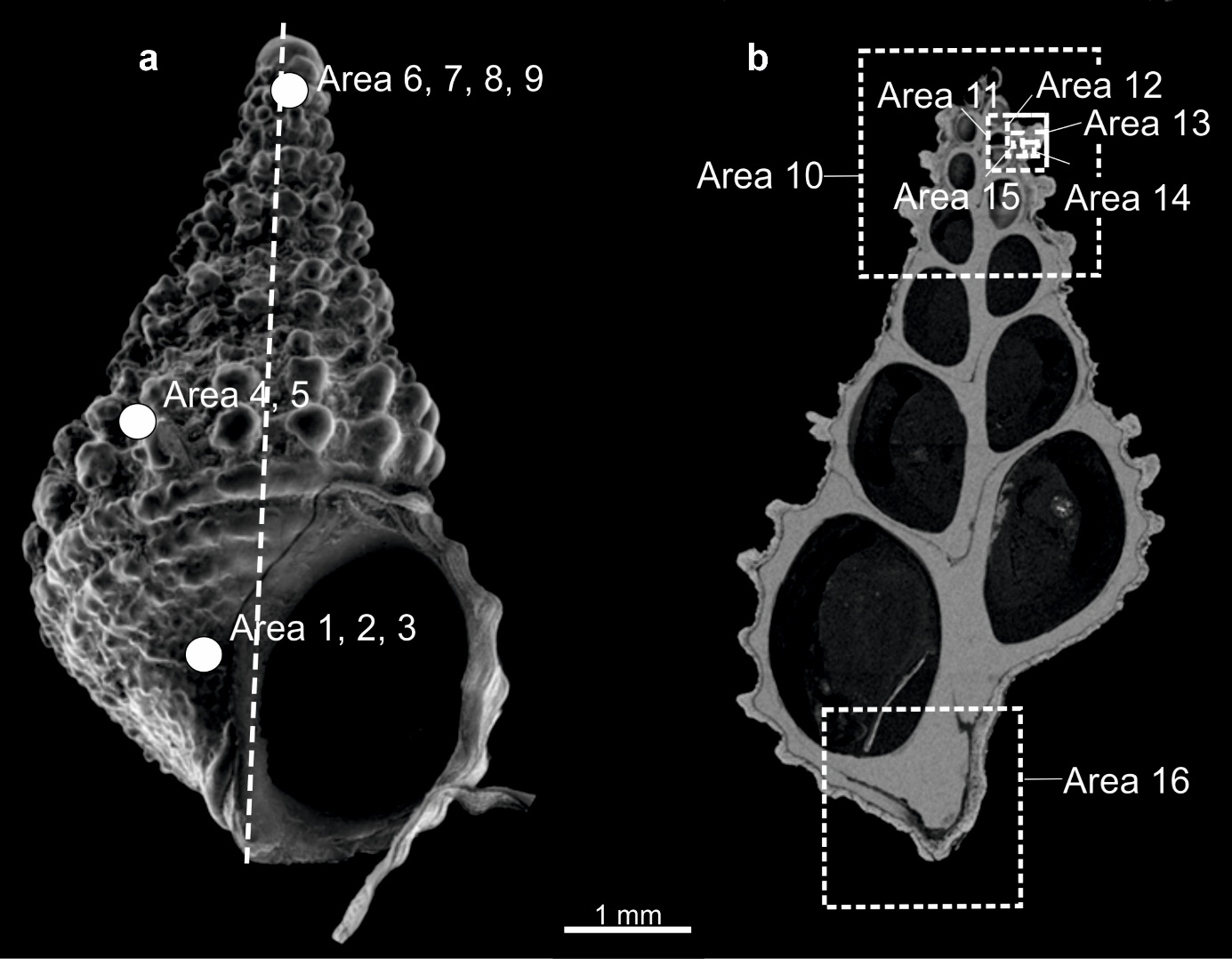


**Area 16**

**Figure S17.** *Desbruyeresia marisindica*, ZPAL Ga.22/4, Kairei Vent Field, Central Indian Ridge; (a) SEM surface view; dashed line indicates the cross-section plane; (b) SEM BSE view of the cross-section. Areas where elemental maps were created on the shell surface and cross-section are indicated.


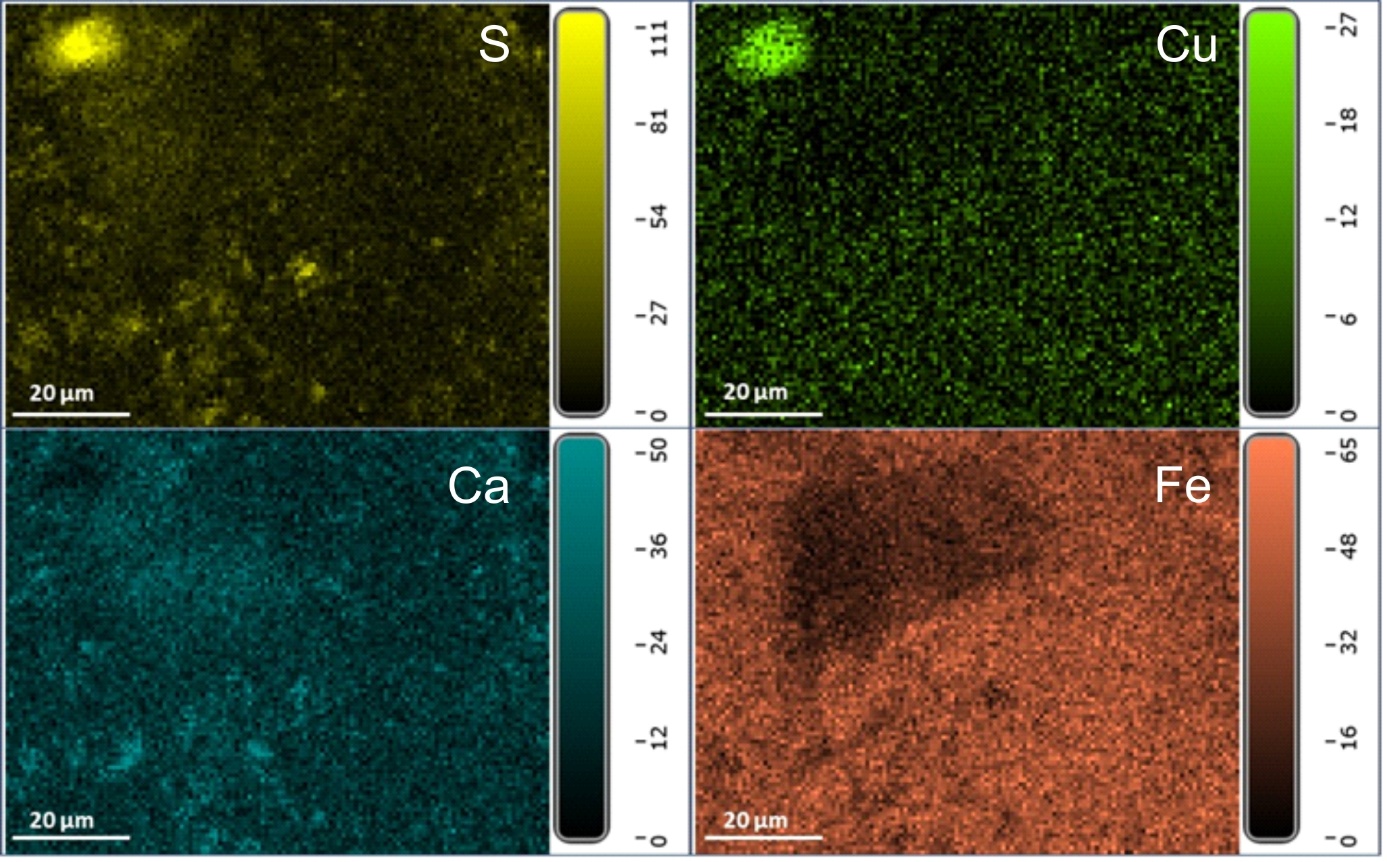


**Figure S18.** EDS elemental maps of the shell surface of *D. marisindica*, ZPAL Ga.22/4, Kairei Vent Field, Central Indian Ridge. Area 9 (See Fig. S17).


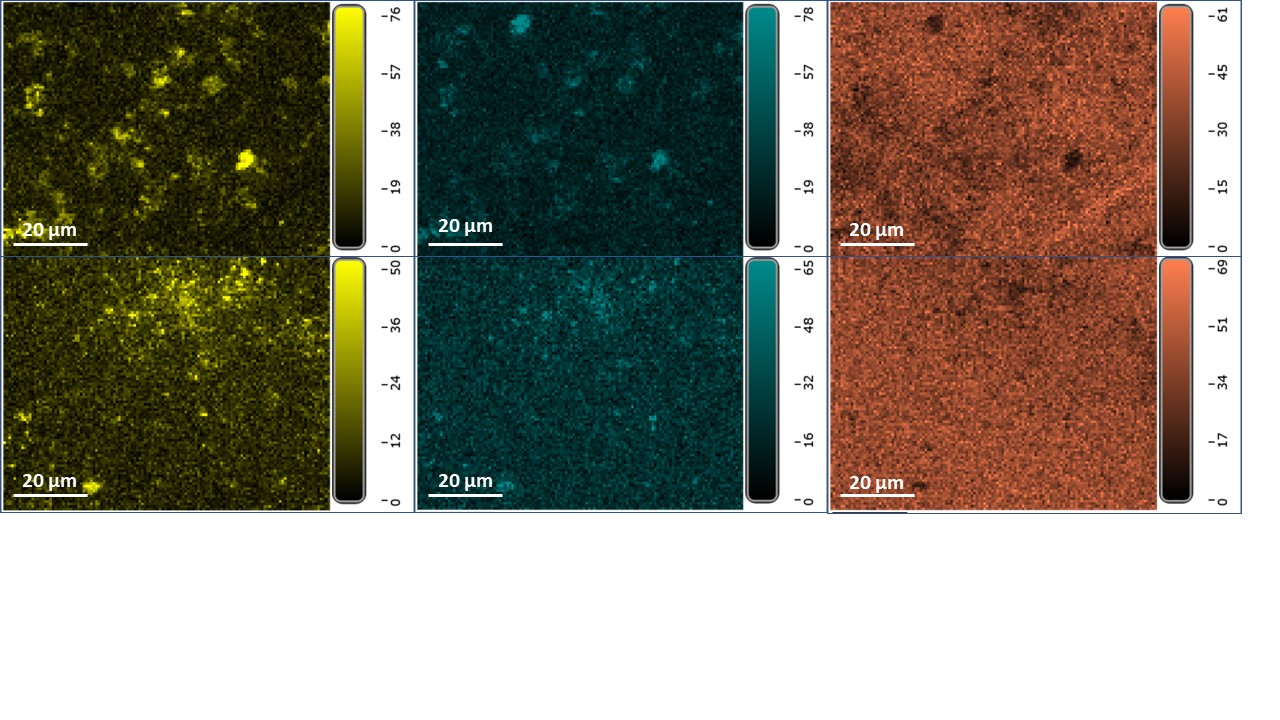


**Fe**

**Fe**

**Ca**

**S**

**S**

**Ca**

**Figure S19.** EDS elemental maps of the shell surface of *D. marisindica*, ZPAL Ga.22/4, Kairei Vent Field, Central Indian Ridge. Top row Area 3; bottom row in Area 6 (see Fig. S17)

**
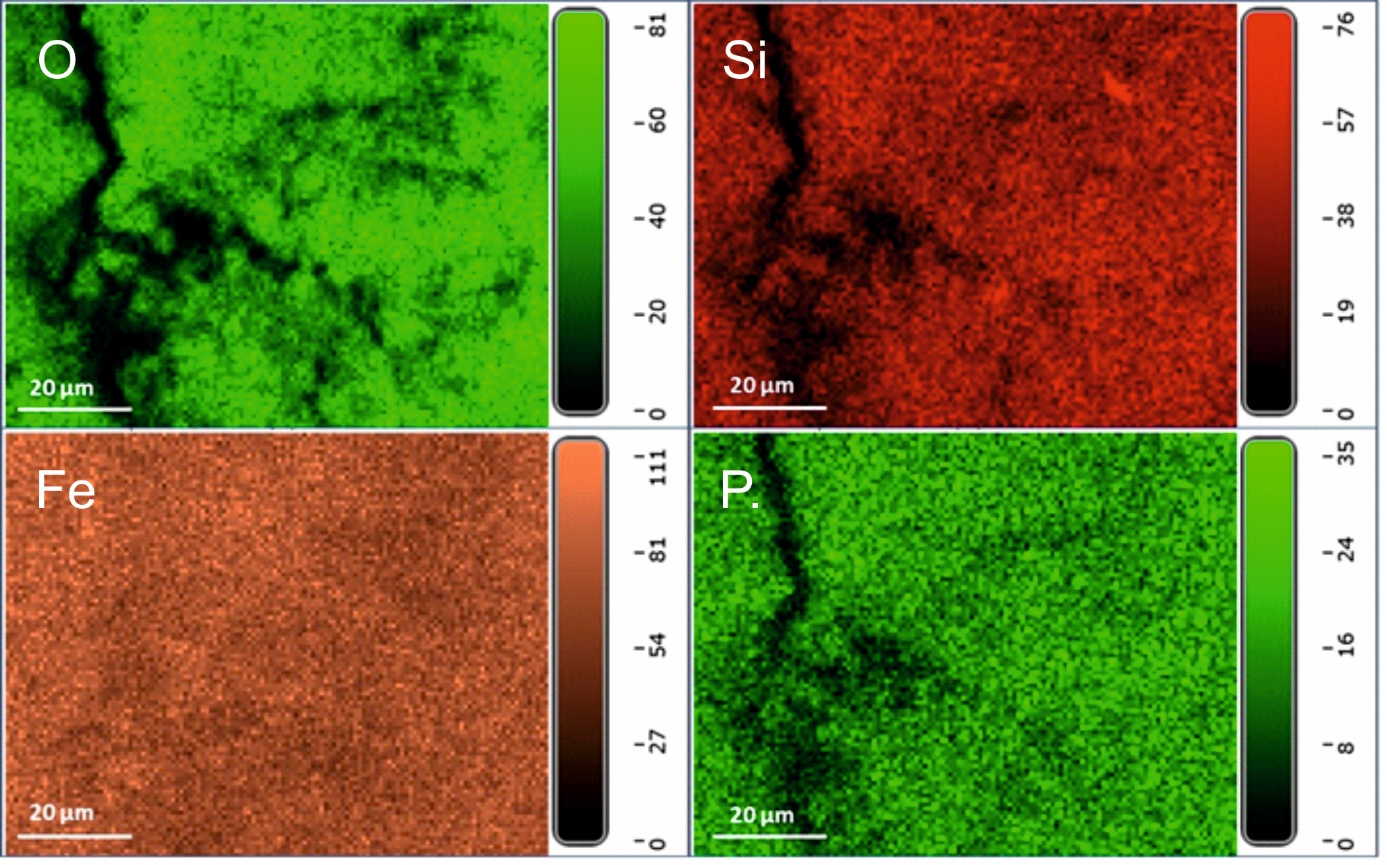
**

**Figure S20.** EDS elemental maps of the cross-section through the shell of *D. marisindica*, ZPAL Ga.22/4, Kairei Vent Field, Central Indian Ridge. Area 16 (see Fig. S17).


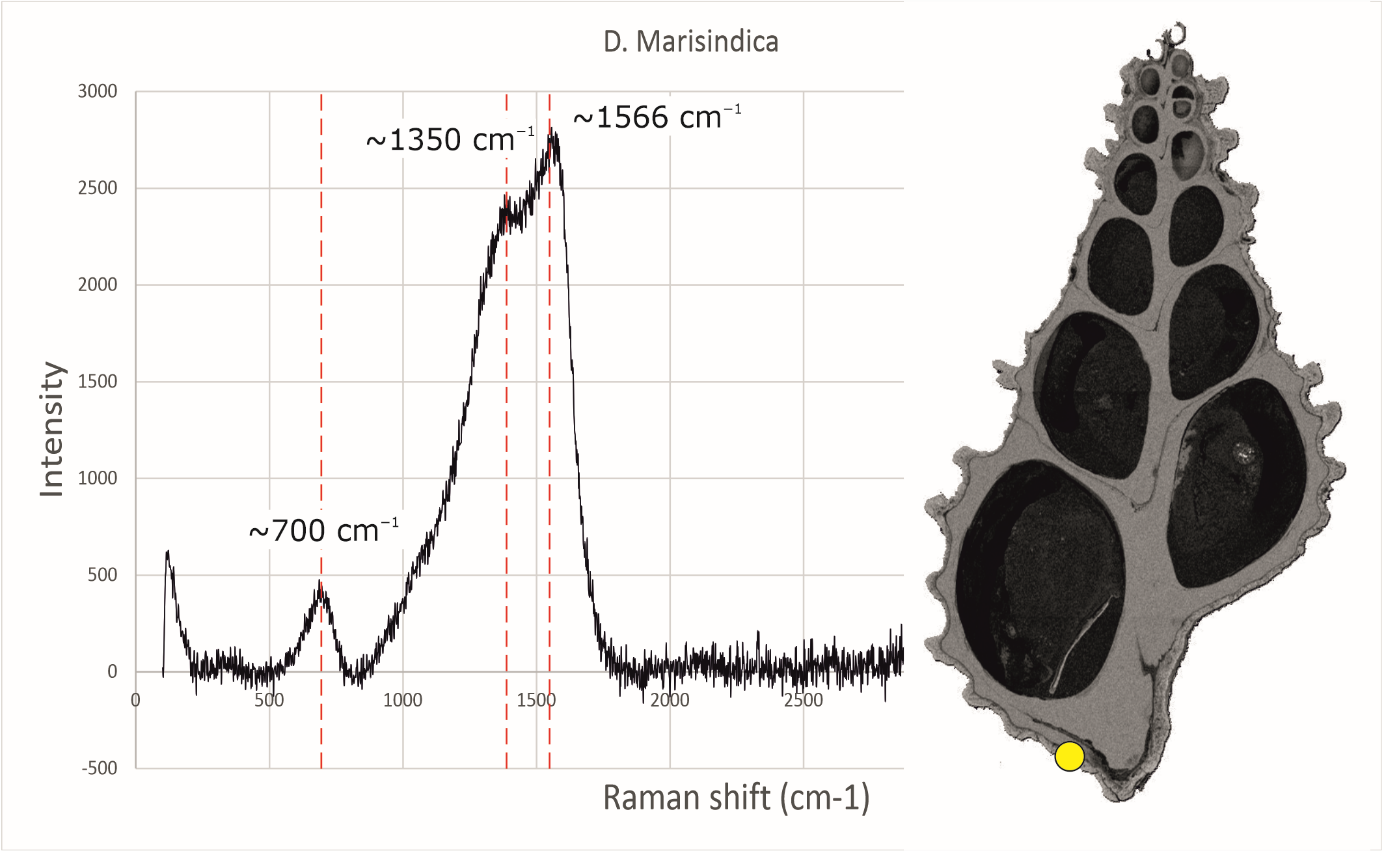


**Fig. S21.** Raman spectrum of mineral coating from *D*. *marisindica*, ZPAL Ga.22/4, Kairei Vent Field, featuring several peaks at 700, 1350, and 1566 cm^−1^. Yellow dot on the right panel highlights the measurement area.


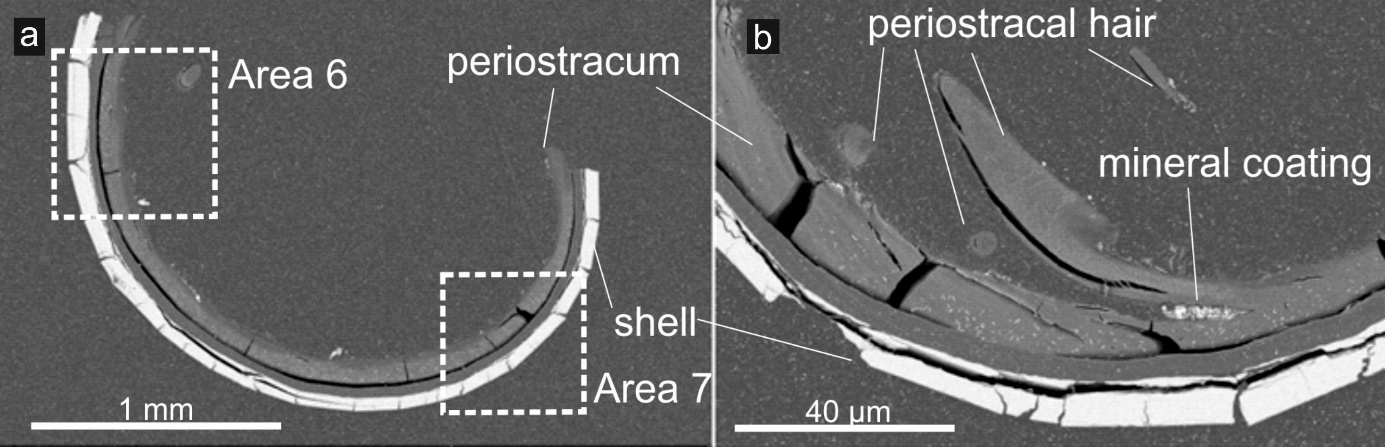


**Figure S22.** SEM BSE images of the two cross-sections through the shell of *Alviniconcha marisindica*, ZPAL Ga.22/5, from the Kairei Vent Field. Areas indicated on (a) were used to create elemental maps of the cross-section; the whole area of (b) was mapped.


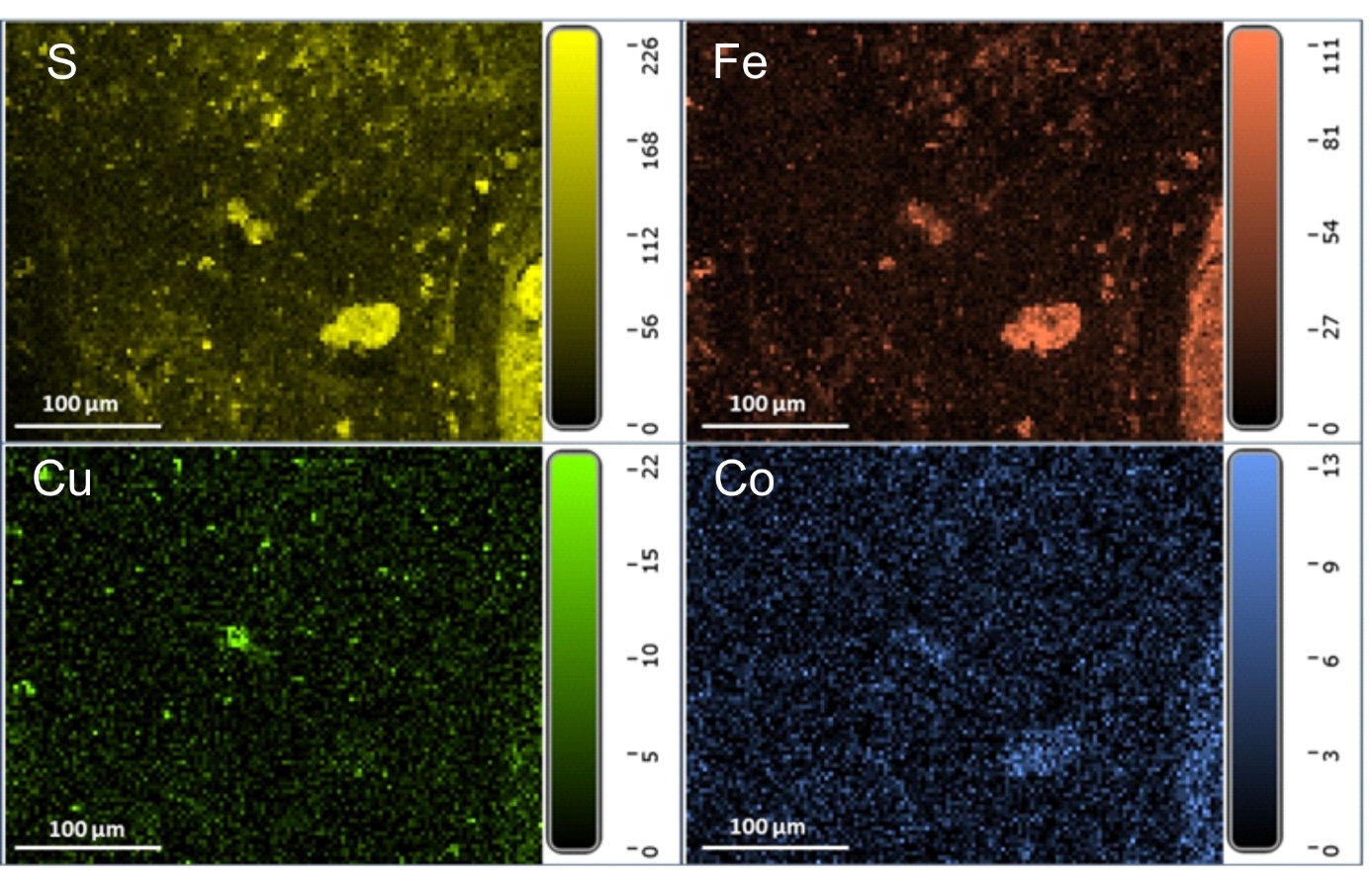


**Figure S23.** EDS elemental maps of the shell surface of *Alviniconcha marisindica*, ZPAL Ga.22/5, Kairei Vent Field, Indian Ocean. Area 2 (see Fig. S22).


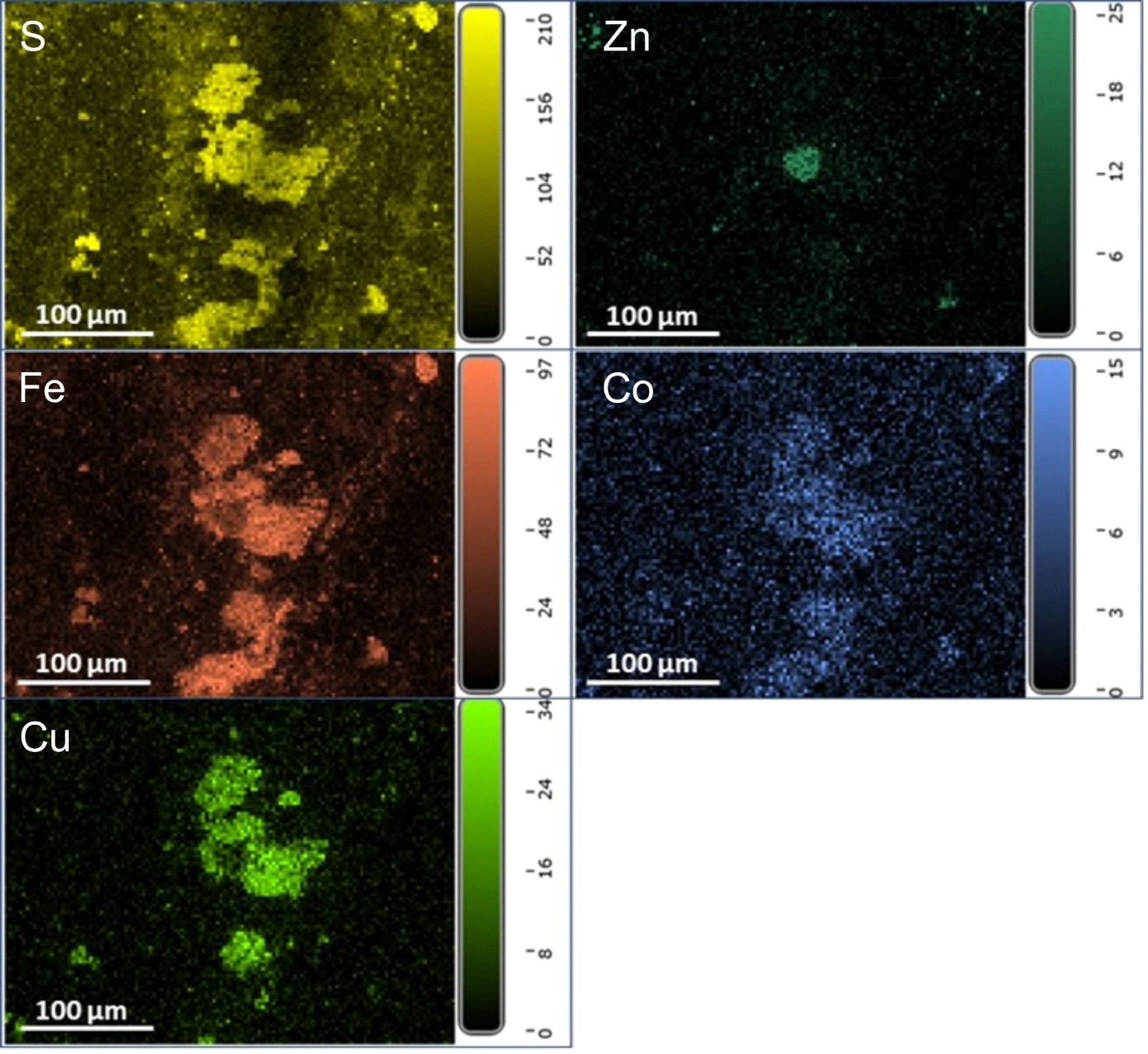


**Figure S24.** EDS elemental maps of the shell surface of *Alviniconcha marisindica*, ZPAL Ga.22/5, Kairei Vent Field. Area 3 (see Fi. S22).


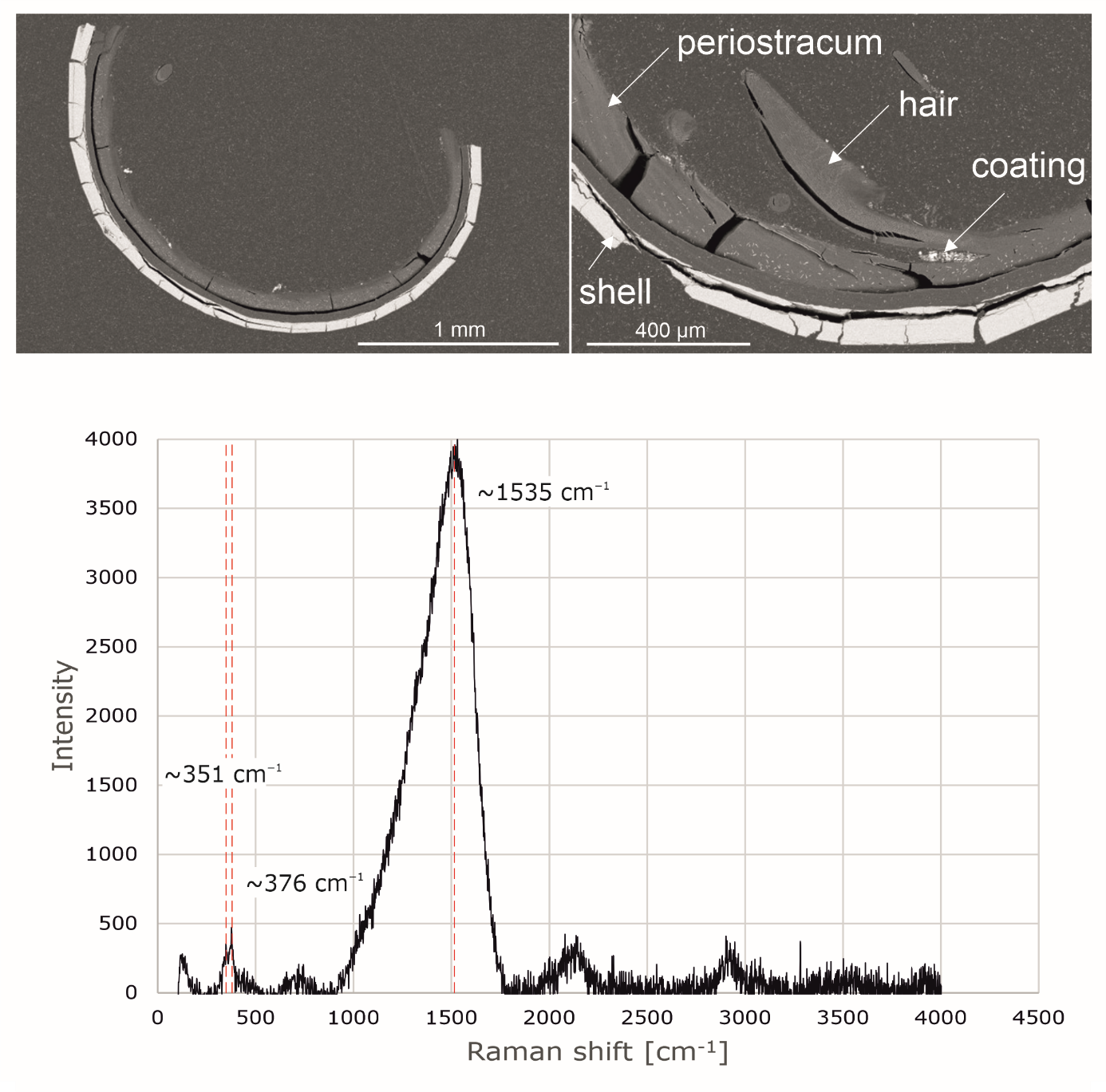


**Fig. S25.** SEM images and Raman spectrum from *A*. *marisindica*, ZPAL Ga.22/5, Kairei Vent Field. Top panels showing the shell, periostracum, mineral coatings, and hairs. Bottom panel shows a Raman spectrum with identifiable peaks at 351, 376, and 1566 cm^−1^ obtained from the coating area shown in the top right panel.


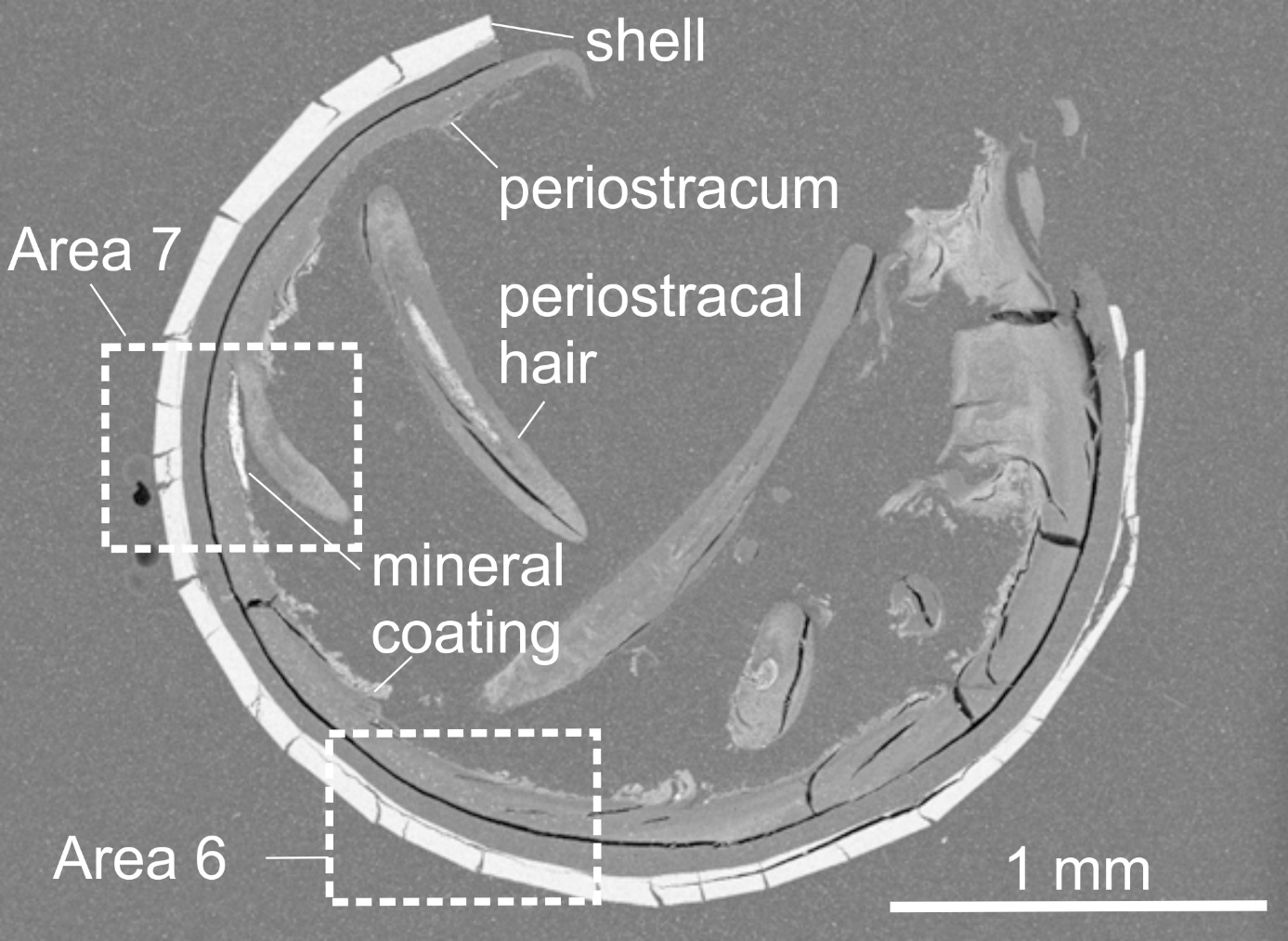


**Figure S26.** SEM BSE images of the cross-section through the shell of *Alvinoconcha marisindica*, ZPAL Ga.22/6, from the Edmond Vent Field; Areas where elemental maps were created on the shell surface and cross-section are indicated.


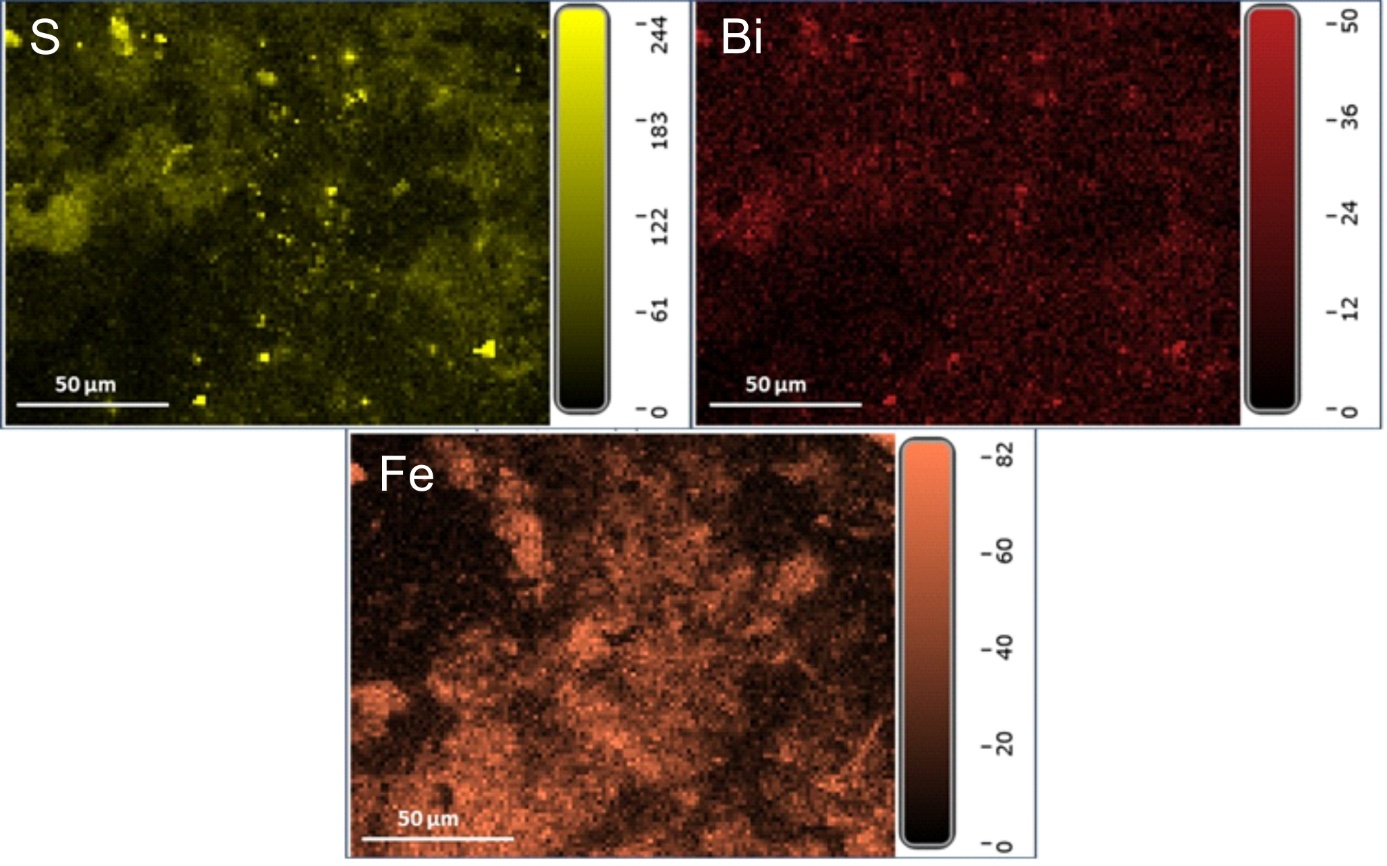


**Figure S27.** EDS elemental maps of the shell surface of *Alvinoconcha marisindica*, ZPAL Ga.22/6, Edmond Vent Field, Central Indian Ocean. Area 5 (See Fig. S26).


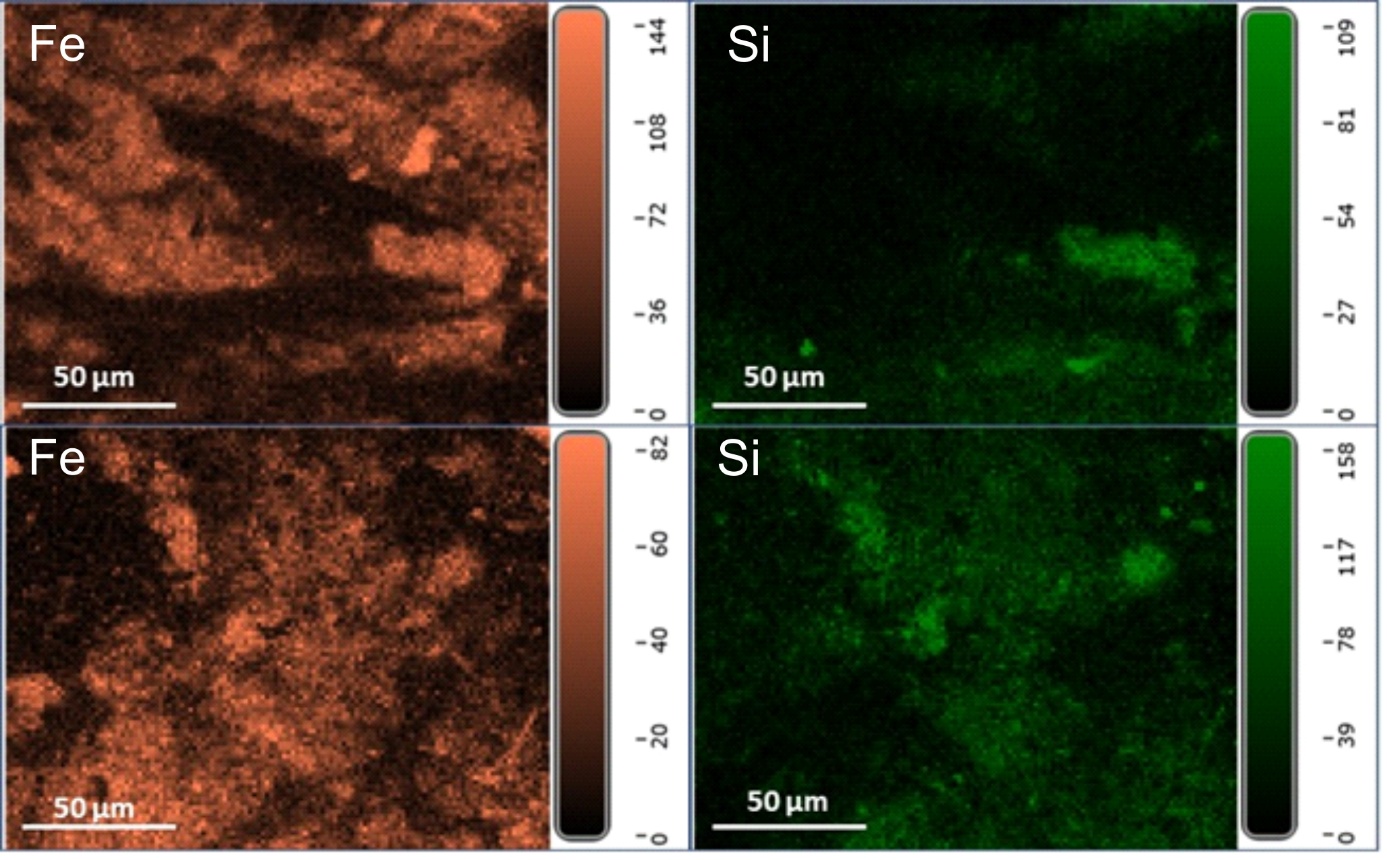


**Si**

**Figure S28.** EDS elemental maps of a periostracal hair of*Alviniconcha marisindica*, ZPAL Ga.22/6, Edmond Vent Field, Central Indian Ocean. Top row in Area 2,; bottom row in Area 5 (see Fig. S26)

**Figure S29.** EDS elemental maps of a periostracal hair of *Alviniconcha marisindica*, ZPAL Ga.22/6, Edmond Vent Field, Central Indian Ocean. Area 4 (see Fig. S26).


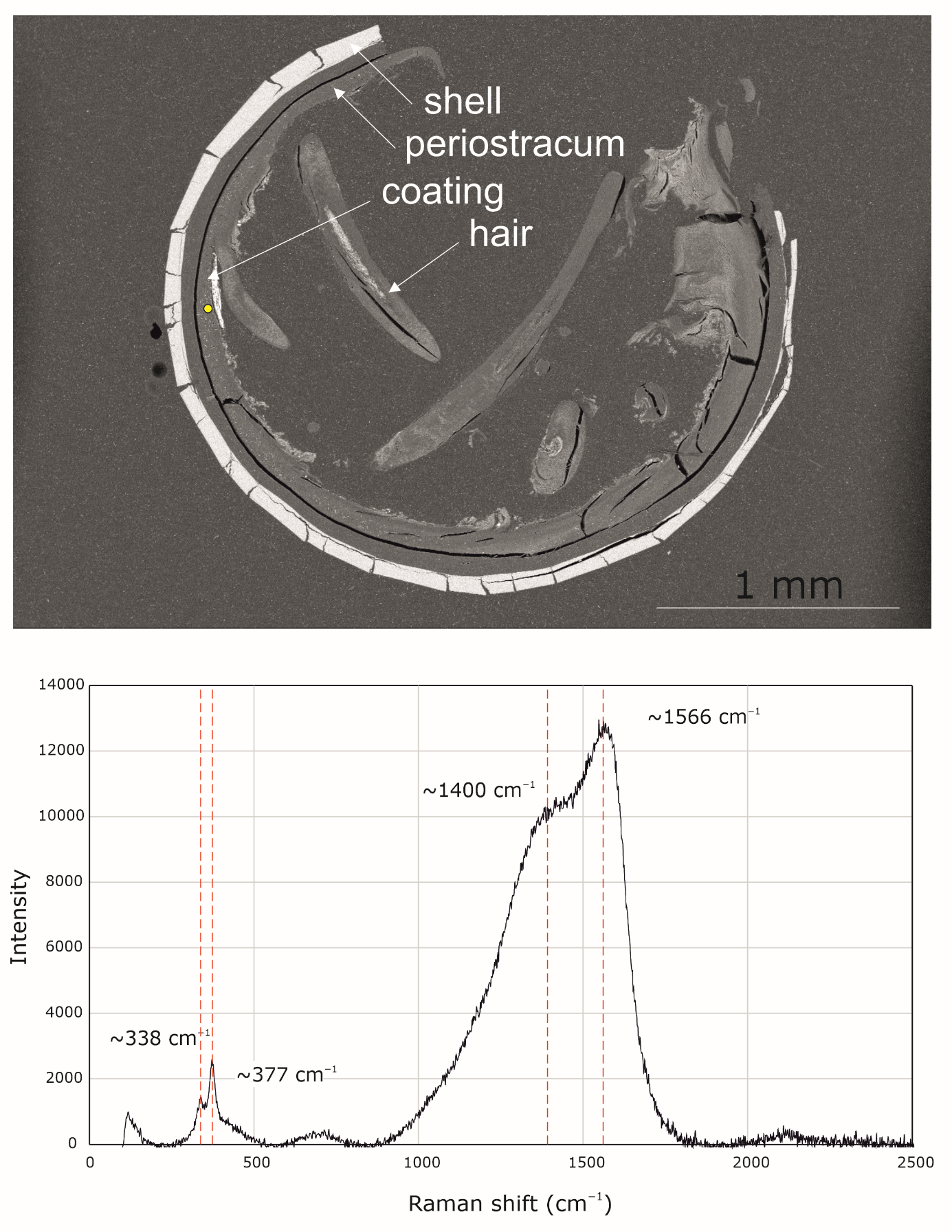


**Fig. S30.** Top panel: REM image of *A*. *marisindica*, ZPAL Ga.22/6, Edmond Vent Field showing shell, periostracum, mineral coating, and hair. The small yellow dot within the coating highlights the area of the Raman measurement. Bottom panel: Raman spectrum from the mineral coating showing distinct peaks between 338 and 377 cm^−1^ , and between 1400 and 1566 cm^−1^.
